# Genomic predictors of response to PD-1 inhibition in children with germline DNA replication repair deficiency

**DOI:** 10.1038/s41591-021-01581-6

**Published:** 2022-01-06

**Authors:** Anirban Das, Sumedha Sudhaman, Daniel Morgenstern, Ailish Coblentz, Jiil Chung, Simone C. Stone, Noor Alsafwani, Zhihui Amy Liu, Ola Abu Al Karsaneh, Shirin Soleimani, Hagay Ladany, David Chen, Matthew Zatzman, Vanja Cabric, Liana Nobre, Vanessa Bianchi, Melissa Edwards, Lauren C, Sambira Nahum, Ayse B. Ercan, Arash Nabbi, Shlomi Constantini, Rina Dvir, Michal Yalon-Oren, Gadi Abebe Campino, Shani Caspi, Valerie Larouche, Alyssa Reddy, Michael Osborn, Gary Mason, Scott Lindhorst, Annika Bronsema, Vanan Magimairajan, Enrico Opocher, Rebecca Loret De Mola, Magnus Sabel, Charlotta Frojd, David Sumerauer, David Samuel, Kristina Cole, Stefano Chiaravalli, Maura Massimino, Patrick Tomboc, David S. Ziegler, Ben George, An Van Damme, Nobuko Hijiya, David Gass, Rose B. McGee, Oz Mordechai, Daniel C. Bowers, Theodore W. Laetsch, Alexander Lossos, Deborah T. Blumenthal, Tomasz Sarosiek, Lee Yi Yen, Jeffrey Knipstein, Anne Bendel, Lindsey M. Hoffman, Sandra Luna-Fineman, Stefanie Zimmermann, Isabelle Scheers, Kim E. Nichols, Michal Zapotocky, Jordan R. Hansford, John M. Maris, Peter Dirks, Michael D. Taylor, Abhaya V. Kulkarni, Manohar Shroff, Derek S. Tsang, Anita Villani, Wei Xu, Melyssa Aronson, Carol Durno, Adam Shlien, David Malkin, Gad Getz, Yosef E. Maruvka, Pamela S. Ohashi, Cynthia Hawkins, Trevor J. Pugh, Eric Bouffet, Uri Tabori

**Affiliations:** 1 Division of Haematology Oncology, The Hospital for Sick Children, Toronto, Ontario Canada; 2 Program in Genetics and Genome Biology, The Hospital for Sick Children, Toronto, Ontario Canada; 3 The Arthur and Sonia Labatt Brain Tumour Research Centre, The Hospital for Sick Children, Toronto, Ontario Canada; 4 Department of Paediatric Haematology/ Oncology, Tata Medical Centre, Kolkata, India; 5 Department of Paediatrics, University of Toronto, Toronto, Ontario Canada; 6 Department of Diagnostic Imaging, The Hospital for Sick Children, Toronto, Ontario Canada; 7 Institute of Medical Science, Faculty of Medicine, University of Toronto, Toronto, Ontario Canada; 8 Princess Margaret Cancer Centre, University Health Network, Toronto, Ontario Canada; 9 Department of Laboratory Medicine and Pathobiology, University of Toronto, Toronto, Ontario Canada; 10 Department of Pathology, College of Medicine, Imam Abdulrahman Bin Faisal University (IAU), Dammam, Saudi Arabia; 11 Department of Biostatistics, Princess Margaret Cancer Center, University Health Network, Toronto, Ontario Canada; 12 Dalla Lana School of Public Health, University of Toronto, Toronto, Ontario Canada; 13 Department of Basic Medical Sciences, Faculty of Medicine, The Hashemite University, Zarqa, Jordan; 14 Department of Medical Biophysics, University of Toronto, Toronto, Ontario Canada; 15 Biotechnology and Food Engineering, Technion – Israel Institute of Technology, Tel-Aviv, Israel; 16 Department of Pediatric Neurosurgery, Dana Children’s Hospital, Tel-Aviv, Israel; 17 Department of Pediatric Hematology–Oncology, Tel-Aviv Sourasky Medical Centre, Tel-Aviv, Israel; 18 Department of Pediatric Hematology–Oncology, Sheba Medical Centre, Ramat Gan, Israel; 19 Department of Paediatric Haematology/Oncology, Centre Hospitalier de Quebec-Universite Laval, Quebec City, Quebec Canada; 20 Departments of Neurology and Pediatrics, University of California, San Francisco, CA USA; 21 Women’s and Children’s Hospital, North Adelaide, South Australia Australia; 22 Department of Pediatrics, University of Pittsburgh School of Medicine, Pittsburgh, PA USA; 23 Neuro-Oncology, Department of Neurosurgery, and Department of Medicine, Division of Hematology/Medical Oncology, Medical University of South Carolina, Charleston, SC USA; 24 Department of Paediatric Haematology and Oncology, University Medical Centre Hamburg–Eppendorf, Hamburg, Germany; 25 Department of Paediatric Haematology–Oncology, Cancer Care Manitoba, Research Institute in Oncology and Haematology, University of Manitoba, Winnipeg, Manitoba Canada; 26 Paediatric Haematology, Oncology and Stem Cell Transplant Division, Padua University Hospital, Padua, Italy; 27 Pediatric Hematology-Oncology, Helen DeVos Children’s Hospital, Grand Rapids, MI USA; 28 Department of Paediatrics, Institute of Clinical Sciences, Sahlgrenska Academy, University of Gothenburg, Sahlgrenska University Hospital, Gothenburg, Sweden; 29 Queen Silvia Children’s Hospital, Sahlgrenska University Hospital, Gothenburg, Sweden; 30 Department of Oncology, Sahlgrenska University Hospital, Gothenburg, Sweden; 31 Department of Paediatric Haematology and Oncology, Second Faculty of Medicine, Motol University Hospital, Charles University, Prague, Czech Republic; 32 Department of Pediatric Oncology, Valley Children’s Hospital, Madera, CA USA; 33 Division of Oncology and Center for Childhood Cancer Research, Children’s Hospital of Philadelphia, Department of Pediatrics, Perelman School of Medicine at the University of Pennsylvania, Philadelpha, PA USA; 34 Paediatric Unit, Fondazione IRCCS Istituto Nazionale dei Tumori, Milan, Italy; 35 Department of Pediatrics, J.W. Ruby Memorial Hospital – West Virginia University, Morgantown, WV USA; 36 Kids Cancer Centre, Sydney Children’s Hospital, Randwick, New South Wales Australia; 37 School of Women’s and Children’s Health, University of New South Wales, Sydney, New South Wales Australia; 38 Division of Hematology and Oncology, Department of Medicine, Medical College of Wisconsin, Milwaukee, WI USA; 39 Department of Paediatric Haematology and Oncology, Saint Luc University Hospital, Université Catholique de Louvain, Brussels, Belgium; 40 Division of Pediatric Hematology/Oncology/Stem Cell Transplantation, Columbia University Irving Medical Centre, New York, NY USA; 41 Atrium Health Levine Children’s Hospital, Charlotte, NC USA; 42 Cancer Predisposition Division, Oncology Department, St Jude Children’s Research Hospital, Memphis, TN USA; 43 Department of Pediatric Hematology Oncology, Rambam Health Care Campus, Haifa, Israel; 44 Department of Pediatrics, The University of Texas Southwestern Medical School, Dallas, TX USA; 45 Department of Oncology, Leslie and Michael Gaffin Center for Neuro-Oncology, Hadassah-Hebrew University Medical Center, Jerusalem, Israel; 46 Neuro-Oncology Service, Tel-Aviv Medical Center, Sackler Faculty of Medicine, Tel Aviv University, Tel-Aviv, Israel; 47 Lux Med Onkologia, Warsaw, Poland; 48 Department of Neurosurgery, Neurological Institute, Taipei Veterans General Hospital, Taipei, Taiwan; 49 Division of Pediatric Hematology/ Oncology/ BMT, Medical College of Wisconsin, Milwaukee, WI USA; 50 Department of Pediatric Hematology-Oncology, Children’s Hospitals and Clinics of Minnesota, St Paul, MN USA; 51 Phoenix Children’s Hospital, Phoenix, AZ USA; 52 Department of Pediatrics, Anschutz Medical Campus, Children’s Hospital of Colorado, Aurora, CO USA; 53 Paediatric Haematology and Oncology, University Hospital Frankfurt, Frankfurt, Germany; 54 Paediatric Gastroenterology, Hepatology and Nutrition Unit, Cliniques Universitaires St Luc, Université Catholique de Louvain, Brussels, Belgium; 55 Children’s Cancer Centre, Royal Children’s Hospital, Murdoch Children’s Research Institute, University of Melbourne, Parkville, Victoria Australia; 56 Department of Paediatrics, University of Melbourne, Parkville, Victoria Australia; 57 Division of Neurosurgery, The Hospital for Sick Children, Toronto, Ontario Canada; 58 Developmental and Stem Cell Biology Program, The Hospital for Sick Children, Toronto, Ontario Canada; 59 Child Health Evaluative Sciences, Research Institute, The Hospital for Sick Children, Toronto, Ontario Canada; 60 Radiation Medicine Program, Princess Margaret Cancer Centre, Toronto, Ontario Canada; 61 Zane Cohen Centre for Digestive Diseases, Mount Sinai Hospital, Toronto, Ontario Canada; 62 Department of Laboratory Medicine and Pathobiology, Faculty of Medicine, University of Toronto, Toronto, Ontario Canada; 63 Massachusetts General Hospital Cancer Center and Department of Pathology, Charlestown, MA USA; 64 Broad Institute of Harvard and MIT, Cambridge, MA USA; 65 Department of Immunology, University of Toronto, Toronto, Ontario Canada; 66 Department of Paediatric Laboratory Medicine, The Hospital for Sick Children, Toronto, Ontario Canada; 67 Program in Cell Biology, The Hospital for Sick Children, Toronto, Ontario Canada; 68 Ontario Institute for Cancer Research, Toronto, Ontario Canada

**Keywords:** Paediatric cancer, Tumour biomarkers, Cancer genetics, CNS cancer, Cancer immunotherapy

## Abstract

Cancers arising from germline DNA mismatch repair deficiency or polymerase proofreading deficiency (MMRD and PPD) in children harbour the highest mutational and microsatellite insertion–deletion (MS-indel) burden in humans. MMRD and PPD cancers are commonly lethal due to the inherent resistance to chemo-irradiation. Although immune checkpoint inhibitors (ICIs) have failed to benefit children in previous studies, we hypothesized that hypermutation caused by MMRD and PPD will improve outcomes following ICI treatment in these patients. Using an international consortium registry study, we report on the ICI treatment of 45 progressive or recurrent tumors from 38 patients. Durable objective responses were observed in most patients, culminating in a 3 year survival of 41.4%. High mutation burden predicted response for ultra-hypermutant cancers (>100 mutations per Mb) enriched for combined MMRD + PPD, while MS-indels predicted response in MMRD tumors with lower mutation burden (10–100 mutations per Mb). Furthermore, both mechanisms were associated with increased immune infiltration even in ‘immunologically cold’ tumors such as gliomas, contributing to the favorable response. Pseudo-progression (flare) was common and was associated with immune activation in the tumor microenvironment and systemically. Furthermore, patients with flare who continued ICI treatment achieved durable responses. This study demonstrates improved survival for patients with tumors not previously known to respond to ICI treatment, including central nervous system and synchronous cancers, and identifies the dual roles of mutation burden and MS-indels in predicting sustained response to immunotherapy.

## Abstract (web summary)

Hypermutation and microsatellite burden determine responses and long-term survival following PD-1 blockade in children and young adults with refractory cancers resulting from germline DNA replication repair deficiency.

## Main

Accurate DNA replication in eukaryotic cells is ensured by the DNA polymerases Pol δ and Pol ε, which control base incorporation and proofreading, and by the mismatch repair (MMR) system that undertakes post-replication surveillance^1^. Germline and somatic mutations in *POLD1* and *POLE* (termed polymerase proofreading deficiency, PPD), or in the MMR genes (*MLH1*, *MSH2*, *MSH6*, *PMS2*; termed MMR deficiency, MMRD*)* result in DNA replication repair deficiency. This is a major driver of hypermutation and microsatellite instability (MSI) in several adult and pediatric cancers^2,3^. Both germline PPD^4^ and monoallelic germline pathogenic variants in the MMR genes (Lynch syndrome)^5^ lead to adult-onset gastrointestinal and genitourinary cancers. In contrast, biallelic loss of MMR function in the germline causes constitutional MMRD syndrome, a highly penetrant and aggressive cancer-predisposing condition. Affected individuals typically develop cancers at a young age, most commonly malignant gliomas, and gastrointestinal and hematological malignancies^6^. These cancers are frequently chemo-resistant and result in poor survival for affected patients. Indeed, these individuals rarely survive beyond early adulthood^6^. The burden is significant in areas of high consanguinity^7^, including many developing countries, and in indigenous populations.

Cancers with DNA replication repair deficiency are universally hypermutant due to the continuous acquisition of multiple somatic mutations. The tumor mutation burden (TMB) of these cancers is 100–1,000-fold higher than MMR-intact pediatric cancers^8^. Furthermore, many of these tumors acquire a secondary somatic mutation in *POLD1/ POLE* leading to combined MMR + PPD, characterised by ultra-hypermutation (>100 mutations per Mb)^2^. As a result, these cancers harbour the highest TMB among all human cancers^8^. Hypermutant cancers such as melanoma^9^ and lung cancer^10^, which are driven by ultraviolet light and smoking, respectively, respond to immune checkpoint inhibitors (ICIs) targeting programmed death 1 (PD-1) signalling. However, despite the dramatic anti-tumor effects reported in several hypermutant adult cancers, these responses are sustained in only a subset of patients^11^. Our understanding regarding the relative importance and variable cut-offs of TMB in determining the nature and duration of response to ICI is still evolving^12–14^. Recent studies have raised questions regarding the roles of TMB and PD-ligand 1 (PD-L1) expression as robust biomarkers of response to ICI^12–15^. In contrast, MMRD colorectal carcinomas are reported to be responsive to ICI due to excess MSI^16–18^, suggesting that genomic features such as TMB or MSI may both drive immune responses to ICI^19–21^, but may not be individually sufficient for durable responses across different cancer types.

Most cancers, including hypermutant adult brain tumors are considered ‘immunologically cold’ and are unresponsive to ICI^22^. Importantly, ICIs did not result in significant responses in multiple large pediatric clinical trials and is considered ineffective in the management of solid tumors in childhood and adolescence^15,23–25^. Additionally, for all solid tumors receiving immunotherapy, the distinction between true tumor progression and an inflammatory pseudo-progression is a major challenge, and a barrier to effective therapy^26^.

Despite the lack of response to ICIs observed in children in previous studies, we hypothesized that cancers originating from germline DNA replication repair deficiency may benefit from ICIs due to their excess mutational load^27^. Furthermore, we postulated that cancers driven by MMRD-only, PPD, or combined MMRD + PPD will respectively exert their own unique mutational spectrum, driving local and systemic immune reactions, which would help shed light on the mechanisms of both response and pseudo-progression following ICI.

To address these hypotheses, we conducted a large, observational, registry-based study, leveraging systematically collected data gathered both retrospectively and prospectively through the International Replication Repair Deficiency Consortium (IRRDC)^2,6,8,27^. This enabled us to evaluate real-world outcomes and predictors of response to anti-PD-1 therapy in children with cancers driven by germline DNA replication repair deficiency. Uniquely, this also provided us the opportunity to investigate the efficacy of ICI in individuals with synchronous malignancies who are otherwise excluded from conventional clinical trials.

## Results

**Fig. 1:**
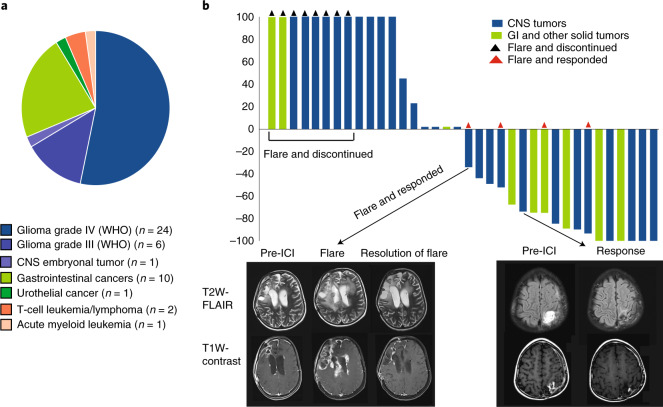
Clinical response to ICI across cancer types in patients with germline DNA replication repair deficiency. **a**, Distribution of tumor types across 38 patients who developed 45 tumors. **b**, Waterfall plot of all radiological responses in non-haematological malignancies. Values show the best fractional change in the 2 dimensions from baseline measurements as per RANO and RECIST criteria (Methods). Arrows point to representative T2-weighted FLAIR and T1-weighted contrast-enhanced MRI sequences in two patients showing flare and partial responses.

Thirty-eight patients who developed 45 cancers were treated with PD-1 inhibitors and followed by the IRRDC study group between May 2015 and March 2019. The PD-1 inhibitor used was either nivolumab (n = 34, 75%) or pembrolizumab (*n* = 11, 25%) (Methods). All patients had germline RRD, diagnosed as constitutional MMRD (*n* = 28, 74%), Lynch (*n* = 8, 21%), or PPD (*n* = 2, 5%) syndromes (Supplementary Table 1). Median age at treatment was 12.1 years (range: 3.1–28.1) for patients with constitutional MMRD, and 15.7 years (range: 8.5–43.4) for those with Lynch syndrome (p = 0.07). Seven cancer types were included and classified into 3 major groups: central nervous system (CNS) tumors (*n* = 31, 69%; disseminated: 2, 6%), non-CNS solid tumors (*n* = 11, 24%; disseminated: 7, 64%), and haematological malignancies (*n* = 3, 7%) (Fig. 1a). The majority (*n* = 43, 93%) of cancers were progressive/recurrent after failure of first-line therapy. Three patients with gastrointestinal cancers received ICI directly following surgery; two had synchronous CNS tumors, and one who had metastatic disease. The data cut-off for outcomes was October 2019.

**Fig. 2:**
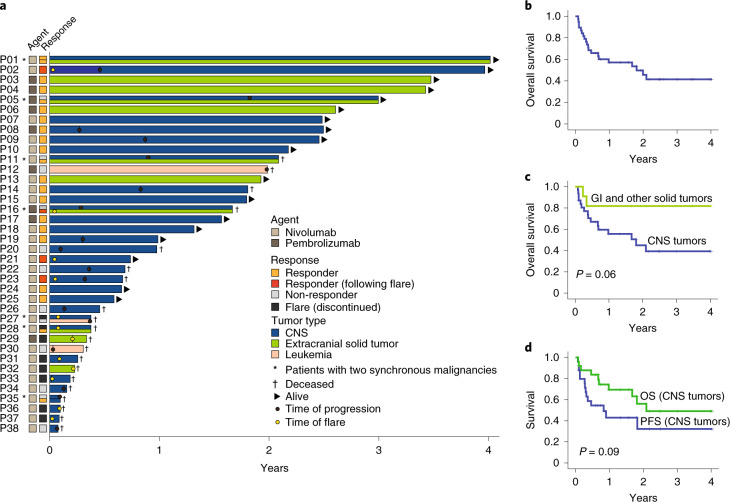
Patient outcome and survival by tumor type. **a**, Swimmer plot by patient and tumor type. **b**, Kaplan-Meier (KM) estimates of overall survival for all patients (**c**) KM estimates of overall survival as per tumor type. Median survival for CNS tumors was 21.6 months. Median survival was not reached for non-CNS solid tumors. Two-sided log-rank test p-value is shown. **d**, KM estimates of progression free and overall survival for CNS tumors continuing ICI therapy. Note: prolonged median survival at 24 months (estimated 3 year OS = 49.1%) despite initial radiological progression at a median of 9.9 months (estimated 3 year PFS = 32%). Two-sided log-rank test p-value is shown.

Responses and/or stable disease were observed in 25/45 (55.5%) tumors, with most of the responses (n = 20; 80%) being sustained at a median follow-up of 1.87 years. Central radiological review (RANO and RECIST criteria; Methods)^28,29^ revealed complete response in 6 (17%), partial response in 9 (25%), stable disease in 7 (19%), and progressive disease in 14 (39%) (Fig. 1b). Among the 7 patients with synchronous malignancies, responses in both tumors were seen in one patient, and at least in one tumor in four patients (Fig. 2a). The three patients with haematological malignancies (leukemia, T-non-Hodgkin lymphoma) progressed at a median time of 4.5 months after starting ICI therapy. Responses were significantly different among the three types of tumors (*P* = 0.0041), with non-CNS tumors having the highest response (100%), followed by CNS tumors (64%), with hematological tumors having the lowest response (0%).

Of note, 12 (27%) tumors exhibited early radiological findings of edema and enhancement, suggestive of peri-tumoral inflammation or tumor progression (Figs. 1b and 2a). This phenomenon occurred at a median of 34 days (range 7–74) from treatment initiation and was termed tumor ‘flare.’^30^ These patients presented with acute clinical deterioration with headache, bone, or abdominal pain, depending on the location of their tumors. Eight patients (6 with CNS and 2 with non-CNS solid tumors) stopped therapy and died. Importantly, 4 patients (3 with CNS and 1 with non-CNS solid tumor) who continued to receive ICI with adequate supportive care subsequently demonstrated objective responses. Since this phenomenon suggested pseudo-progression, these tumors were studied in more detail.

Estimated 3 year overall survival (OS) was 41.4% (95% CI: 38.5–44.2) (Fig. 2b), with 18 (47%) patients being alive at the time of last follow-up (Fig. 2a). This is noteworthy considering the refractory nature of their cancers. Analysis by cancer type revealed that non-CNS solid tumors had a significantly better survival compared to CNS tumors (Fig. 2c and Supplementary Fig. 1d; *P* = 0.01). Nevertheless, the OS of 39.3%, (95% CI: 36.3–42.3) and PFS of 26.9% (95% CI; 23.2, 30.6) for patients with recurrent/progressive CNS tumors is a dramatic improvement compared to their historically rapidly fatal outcomes (Fig. 2c,d and Supplementary Fig. 1b)^6,7,27^. All patients with non-CNS solid tumors continuing ICI had durable responses and are alive at a median follow-up of 2.6 years (range: 0.38–3.5). Remarkably, 13 patients with CNS tumors who experienced radiological progression on initial ICI therapy had prolonged survival (median, 9.6 months; range, 1.5–27 months) (Fig. 2a,d and Supplementary Table 1). Plausible explanation for the late and continued responses to immunotherapy is the obligatory mutation accumulation in these cancers^8^, which result in novel immunogenic neoantigens and responses. Clinical variables such as age, gender, ethnicity, prior treatment, or choice of ICI agent were not associated with outcome (Supplementary Fig. 2).

### Molecular determinants of response to immunotherapy

**Fig. 3:**
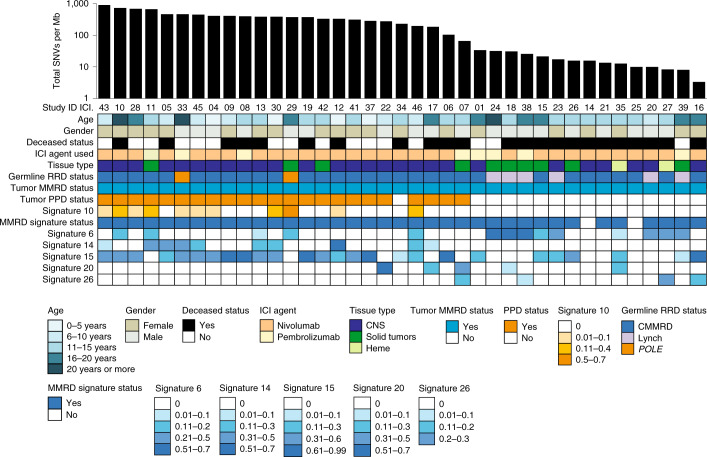
Onco-plot summarising the genomic features from 39 available paired tumors and germline exomes, and their clinical correlates. The tumors are arranged in descending order by total single nucleotide variants (SNVs) per megabase using semi-logarithmic scale. Tumor status for mismatch repair deficiency (MMRD) was determined based on MMR gene mutation status by whole exome sequencing (WES), or genetic testing of the patient germline. Tumor polymerase-proofreading deficiency (PPD) status was ascertained by presence of known *POLE/POLD1* driver mutations and/or COSMIC signature status for PPD (COSMIC signature 10). The relative weight of each mutational signature contributing to each specific tumor sample analysis as reported using the DeconstructSigs package (Methods) has been color-coded, with the values provided below the onco-plot.

**Fig. 4:**
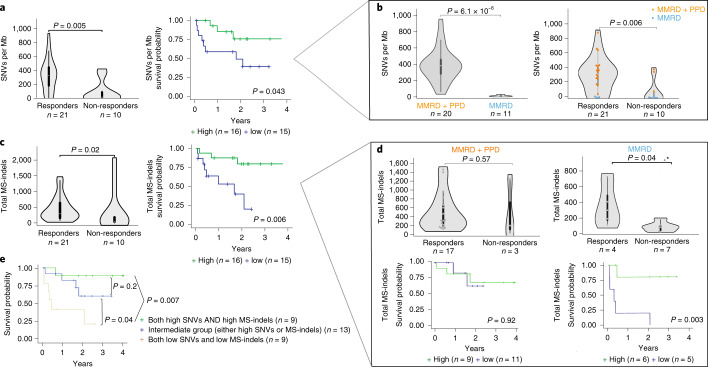
Genomic biomarkers, survival and response to PD1 blockade. **a**, Response and overall survival (OS) by single nucleotide variants (SNVs) per Mb. For survival analysis, median SNV burden was used. **b**, SNVs as a function of MMRD (blue) and MMRD + PPD (orange) status (left), and response association with both SNV and replication-repair deficiency status. **c**, Response and overall survival (OS) by microsatellite indels (MS-indels). For survival analysis, median MS-indel values were used. **d**, Response and overall survival by total MS-indel count for MMRD + PPD and MMRD-only cancers separately. **e**, Kaplan-Meier (KM) estimates using combined SNVs/Mb and MS-indel in all replication repair-deficiency cancers. For all box-plots for responders and non-responders, data are represented as median + /- interquartile range. For statistical significance in comparing responders and non-responders, the Wilcoxon-Mann-Whitney test was used. Survival analysis was performed using the Kaplan-Meier method, and the log-rank test was used to compare groups. All p values are 2-sided. (MMRD: mismatch repair deficiency; PPD: polymerase proofreading deficiency; MS-indel: microsatellite insertion/ deletion).

To better understand the molecular determinants of response to ICI, biopsy specimens and blood samples were collected before and during therapy from the patients for centralized analysis (Methods). Whole exome analysis of tumors (*n* = 39, Fig. 3) revealed high variability in the number of single nucleotide variants (SNV), including ultra-hypermutation (median, 233.8 mutations per Mb; range, 3.4–912), which was associated with tumor genotype. MMRD-only cancers (*n* = 16) had significantly fewer SNVs (median, 15.8 mutations/Mb) than MMRD + PPD cancers (*n* = 23, median 391.4 mutations per Mb; *P* < 0.0001; Fig. 4b). This was associated with germline status, given that 21 (67.7%) cancers in constitutional MMRD patients harboured MMRD + PPD (median, 398.98 mutations per Mb), while all cancers in individuals with Lynch syndrome lacked somatic PPD (median, 21.76 mutations per Mb; *P* = 0.03). Both cancers originating from germline PPD, one colorectal carcinoma (P13; ICI.29) and one glioblastoma (P17; ICI.33) had acquired somatic MMRD resulting in ultra-hypermutation (Figs. 2a and 3).

Analysis of COSMIC signatures, which reflect the imprints of the underlying mutational processes^31^, revealed that signature 6 was enriched in tumors from germline Lynch syndrome patients (*P* = 0.003), whereas signatures 10 and 14 were not detected in these tumors. Furthermore, signature 6 was enriched in MMRD-only cancers (*P* = 0.04), and signatures 10 and 14 were enriched in MMRD + PPD cancers (*P* < 0.002). Last, signatures 10 and 14 were individually associated with response to ICI (*P* = 0.03). Importantly, signature 11, which is commonly detected in treatment-related hypermutant adult gliomas that do not respond to ICI^32^, was detected only in a single patient (P27) with synchronous leukemia and glioblastoma, who had been previously treated with alkylating agents for medulloblastoma. These findings highlight the unique diagnostic and prognostic roles of mutational signatures in replication repair-deficient cancers^31^.

**Extended Data Fig. 1:**
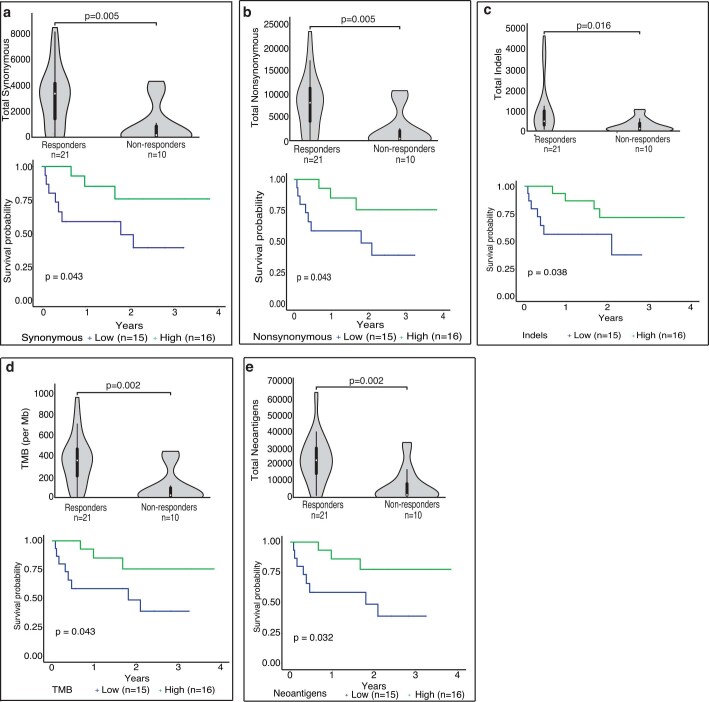
Genomic biomarkers (SNVs), survival and response to PD-1 blockade. Response and overall survival by (a) total synonymous variants, (b) non-synonymous variants, (c) total indels, (d) total mutations/Mb, and (e) neoantigens. For survival, median values were utilized to stratify into ‘high’ and ‘low’ groups. For all box-plots for responders and non-responders, data are represented as median ± interquartile range. For statistical significance in comparing responders and non-responders, the Wilcoxon-Mann-Whitney test was used. Survival analysis was performed using the Kaplan-Meier method, and the log-rank test was used to compare groups. All p values are 2-sided.

**Extended Data Fig. 2:**
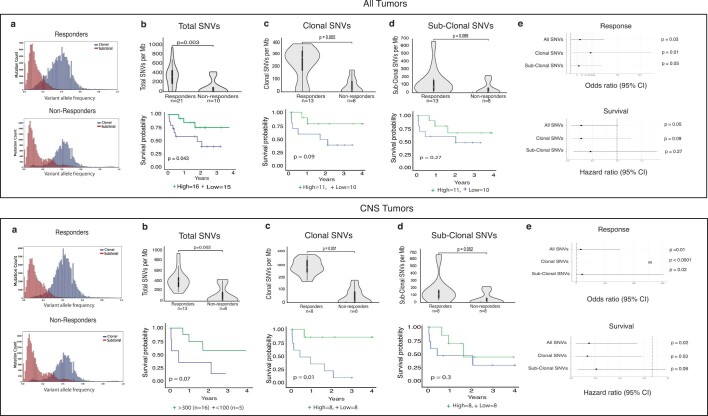
Clonal SNVs and outcomes. (a) Distribution (using histogram) of clonal versus sub-clonal mutations between responders and non-responders for all (above) and CNS tumors (below). (b-d): Response and survival (stratification by >= median) for total, clonal and sub-clonal SNVs. For all box-plots for responders and non-responders, data are represented as median +/- interquartile range. (e) Odds ratio (95% confidence interval) for response (using logistic regression) and hazard ratios (95% confidence interval) for survival (using Cox regression) for all, clonal and sub-clonal SNVs for all tumors (above) and CNS tumors (below). All p-values are 2 sided.

As questions exist regarding the contribution of tumor-intrinsic characteristics such as mutation load in terms of SNVs, indels, and the microsatellite specific indels to ICI response, we sought to determine whether independent roles exist for each of these genomic features and their underlying driver mechanisms. High SNV/Mb (≥median: 275.38/Mb) was significantly associated with both response and survival, demonstrating that extreme mutation burdens were relevant even for a cohort of uniformly hypermutant cancers (*P* = 0.005, Fig. 4a and Extended Data Fig. 1a–d). Both response and survival were also associated with a higher tumor neoantigen load (Extended Data Fig. 1e). Enrichment of clonal mutations^33^ predicted response (Extended Data Fig. 2). Remarkably, response and survival were predicted by replication repair deficiency status. Patients whose cancers were MMRD + PPD had higher mutation burden (*P* < 0.0001), and these tumors were enriched among the responders (*n* = 17/21) as compared to non-responders (*n* = 3/10) (*P* < 0.001) (Fig. 4b).

**Extended Data Fig. 3:**
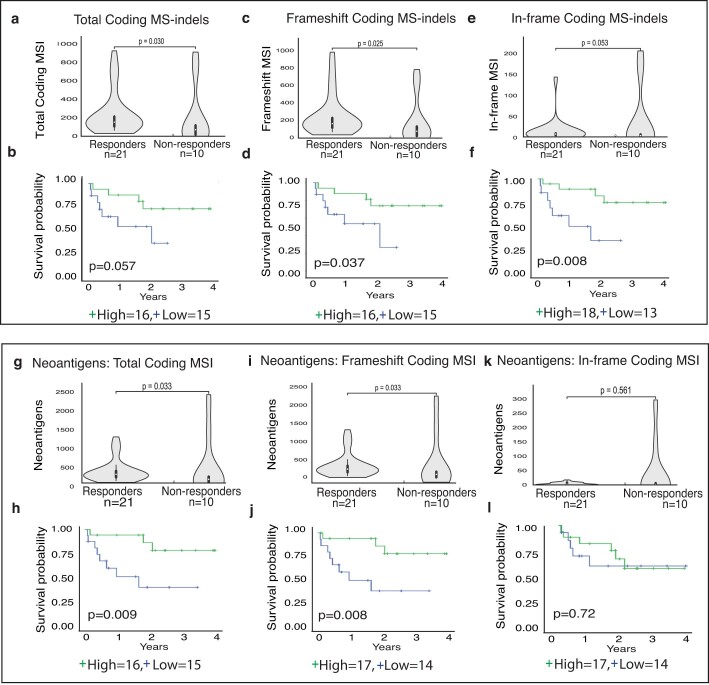
Genomic biomarkers (MS-Indels), survival and response to PD-1 blockade. (a-f) Total, frameshift and in-frame MS-Indels and outcome. (g-l) Neoantigens generated by total, frameshift and in-frame MS-Indels and outcome. For survival, median values were utilized to stratify into ‘high’ and ‘low’ groups. For all box-plots for responders and non-responders, data are represented as median +/- interquartile range. For statistical significance in comparing responders and non-responders, the Wilcoxon-Mann-Whitney test was used. Survival analysis was performed using the Kaplan-Meier method, and the log-rank test was used to compare groups. All p values are 2-sided.

Recent work has shown that the degree of genome-wide MS-indels correlate with response to ICI in MMRD tumors^3,34,35^. Indeed, across our entire cohort, total MS-indels, calculated by MS-mutect (Methods)^36^, were predictive of tumor response (*P* = 0.02) and patient survival (p = 0.006; Fig. 4c). This was as a result of frameshift indels in coding microsatellites and neoantigens generated by these MS-indels, which strongly predicted outcome (Extended Data Fig. 3).

**Extended Data Fig. 4:**
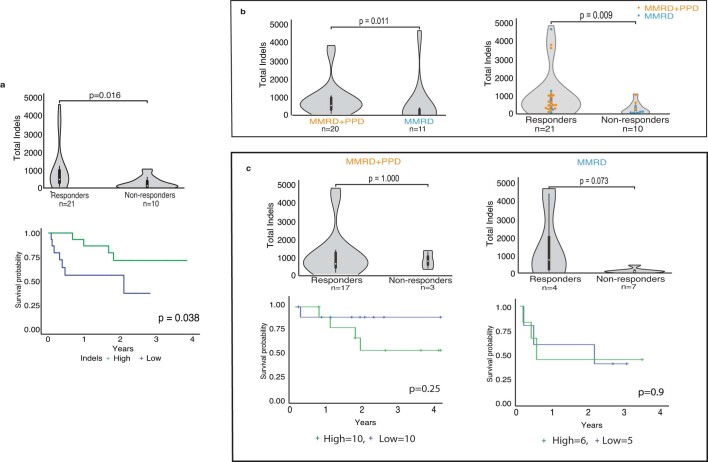
Genomic biomarkers (Total Indels), survival and response to PD1 blockade. (a) Response and overall survival (OS) by total indels. For survival analysis, median indel burden was used. (b) Total indels as a function of MMRD (blue; n=11) and MMRD+PPD (orange; n=20) status (left), and response association (responders; n=21; non-responders; n=10) with both indels and replication-repair deficient status. (c) Response and overall survival (OS) by total indels, stratified by tumors with MMRD+PPD (n=20) and MMRD only (n=11). For survival analysis, median indel burden was used. For all box-plots for responders and non-responders, data are represented as median +/- interquartile range. For statistical significance in comparing responders and non-responders, the Wilcoxon-Mann-Whitney test was used. Survival analysis was performed using the Kaplan-Meier method, and the log-rank test was used to compare groups. All p values are 2-sided.

Given that no significant correlation between MS-indels and SNVs were observed (Supplementary Fig. 3), we hypothesized that while both SNVs and MS-indels are independently immunogenic across all replication repair-deficient cancers, MS-indels may be particularly important for determining outcomes for MMRD-only cancers which harbour relatively lower SNVs. To test this, we analyzed MMRD-only and MMRD + PPD tumors separately (Fig. 4d and Supplementary Fig. 4). Total MS-indels were higher in responders than in non-responders and were significantly associated with survival in MMRD-only cancers (*P* = 0.003) and not in MMRD + PPD cancers (Fig. 4d). In contrast, high SNVs and total indels failed to predict response in MMRD-only cancers (*P* > 0.1, Extended Data Fig. 4c and Supplementary Fig. 4b). Combining the prediction models for both these types of replication repair deficiency cancers (MMRD-only and MMRD + PPD) revealed that high SNVs and total MS-indels together strongly predicted improved survival (Fig. 4e and Supplementary Fig. 3d–g; *P* = 0.0024).

**Extended Data Fig. 5:**
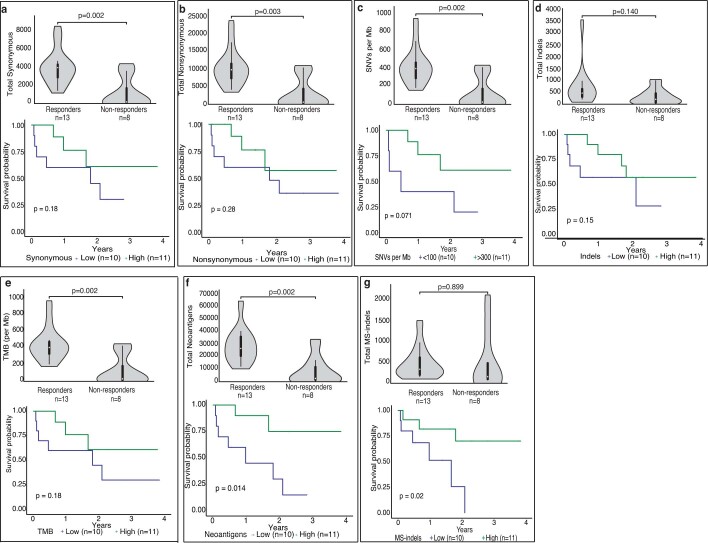
CNS tumours: Genomic biomarkers, survival and response to PD-1 blockade. Response and overall survival for CNS tumours by (a) synonymous variants, (b) non-synonymous variants, (c) SNVs/Mb, (d) total indels, (e) total mutations/ Mb, (f) neoantigens, and (g) MS-indels. For survival, median values were utilized to stratify into ‘high’ and ‘low’ groups. For all box-plots for responders and non-responders, data are represented as median +/- interquartile range. For statistical significance in comparing responders and non-responders, the Wilcoxon-Mann-Whitney test was used. Survival analysis was performed using the Kaplan-Meier method, and the log-rank test was used to compare groups. All p values are 2-sided.

To determine whether mutations and MS-indels drive responses within a more homogeneous cancer type, we interrogated both these genomic markers in CNS tumors. Both components independently contributed to the response and survival (Extended Data Fig. 5) and no differences in these genomic features were observed between CNS and non-CNS solid tumors (Supplementary Fig. 5a–d). Importantly, clonal mutations^33^ were particularly a strong predictor of response and survival for CNS tumors (Extended Data Fig. 2).

Collectively, these data suggest dual roles for SNVs and MS-indels in determining immunotherapy responses in replication repair-deficient cancers (Supplementary Fig. 3d,g), including CNS tumors (Supplementary Fig. 6).

### Immune microenvironment and response to therapy

Next, we examined whether the tumor subgroups affect the tumor micro-environment and response to therapy. We tested multiple immune markers using immunohistochemistry for immune cell infiltration (CD3, CD4, CD8 and CD68) and checkpoint ligand (PD-L1) expression. All immune markers were scored independently by two pathologists with robust concordance (Methods; Supplementary Fig. 7).

**Fig. 5:**
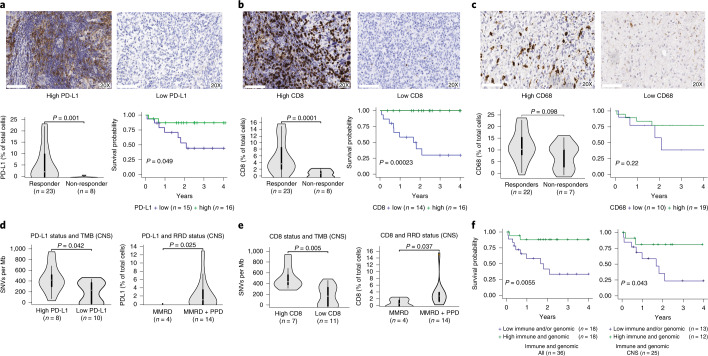
Tumor immune microenvironment, survival and response to PD-1 blockade. **a**, PD-L1 expression, response and survival in all replication repair-deficient cancers. Cut-off is ≥ 1% of cells (median; Methods). **b**, CD8 expression, response and survival for replication repair-deficient cancers. Cut-off is ≥ 3% of cells (median; Methods). **c**, CD68 expression, response and survival. Cut-off is ≥ 12.5% of cells (median; Methods). For (**a**), (**b**) and (**c**), the histology depicts glioblastoma at 20X magnification. **d**,**e**, Association of immune markers with SNV and replication repair-deficient status. **f**, Combined immune (PD-L1 and CD8 expression) and genomic (TMB and MS-indels) and overall survival in replication repair-deficient cancers. For all box-plots for responders and non-responders, data are represented as median + /- interquartile range. For statistical significance in comparing responders and non-responders, the Wilcoxon-Mann-Whitney test was used. Survival analysis was performed using the Kaplan-Meier method, and the log-rank test was used to compare groups. All p values are 2-sided.

**Extended Data Fig. 6:**
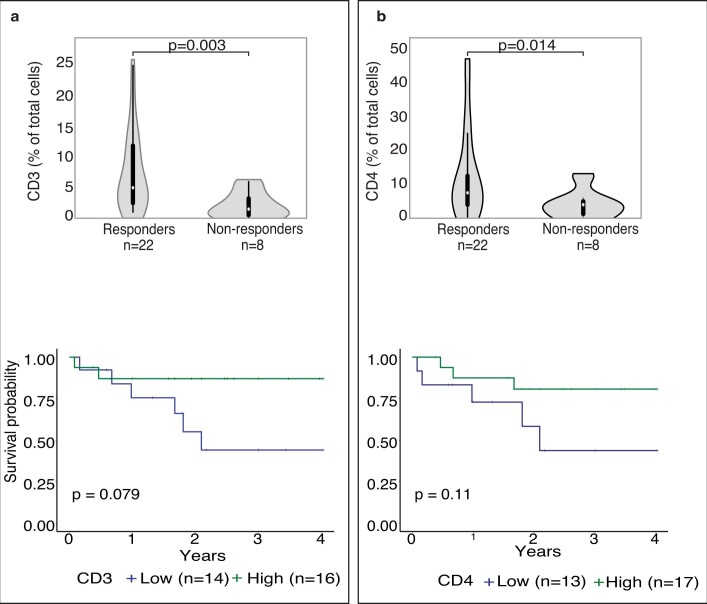
Tumour immune microenvironment, survival and response to PD-1 blockade. Response and overall survival by (a) CD3, and (b) CD4-positive cells in the pre-ICI tumour specimens. For survival, median values were utilized to stratify into ‘high’ and ‘low’ groups. For all box-plots for responders and non-responders, data are represented as median +/- interquartile range. For statistical significance in comparing responders and non-responders, the Wilcoxon-Mann-Whitney test was used. Survival analysis was performed using the Kaplan-Meier method, and the log-rank test was used to compare groups. All p values are 2-sided.

**Extended Data Fig. 7:**
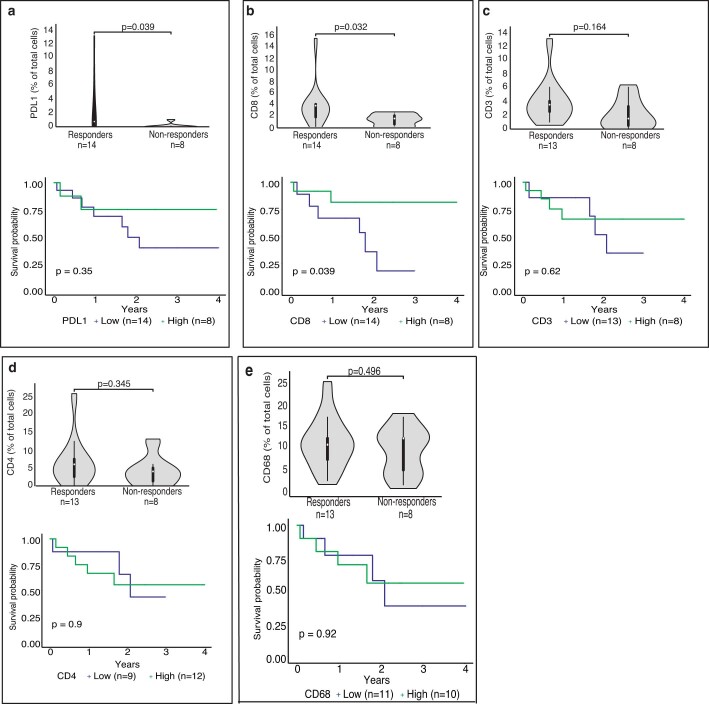
CNS tumours: Immune microenvironment, survival and response to PD-1 blockade. Response and overall survival in CNS tumours by (a) PD-L1, (b) CD8, (c) CD3, (d) CD4, and (e) CD68 expression. For survival, median values were utilized to stratify into ‘high’ and ‘low’ groups. For all box-plots for responders and non-responders, data are represented as median +/- interquartile range. For statistical significance in comparing responders and non-responders, the Wilcoxon-Mann-Whitney test was used. Survival analysis was performed using the Kaplan-Meier method, and the log-rank test was used to compare groups. All p values are 2-sided.

PD-L1 expression was associated with both response and improved survival (*P* = 0.04, Fig. 5a). Overall, increased lymphocytic infiltration within the tumor microenvironment was associated with response (Extended Data Fig. 6). Specifically, high CD8-T cell infiltration predicted both response and improved OS (*P* = 0.0002, Fig. 5b). All non-CNS solid tumors including those with MMRD-only, harboured high MSI, exhibited high CD8+ T cell infiltration (Supplementary Fig. 5), and responded to ICI. This corroborates previous reports in which MMR-deficient gastrointestinal tumors had high CD8+ T cell infiltration^37^. High T cell infiltration was also observed in CNS tumors, which are traditionally considered an ‘immune-privileged’ site^22^ (Fig. 5b). CNS tumors with a high mutation burden and MMRD + PPD not only had increased CD8+ T cell infiltration, but also significantly higher expression of PD-L1 (Fig. 5d,e). In contrast, all tumors in our study had low to moderate CD68 expression (none > 30%). Furthermore, there was no association between CD68 and response or survival (Fig. 5c). This suggests that in the setting of ultra-hypermutation driven by combined MMRD + PPD and high genomic MS-indels, the increased activation of the immune microenvironment, associated with robust CD8+ T cell responses, can explain the remarkable responses seen even in the CNS tumors. Indeed, in these CNS tumors, high CD8+ T cell infiltration was associated with improved OS (*P* = 0.039; Extended Data Fig. 7b).

Overall, tumors exhibiting high expression of immune markers and a favorable genomic profile (high SNVs or MS-indels) had a 3 year OS of 87.8% (95% CI: 84–91.5) as compared to 33.2% (95% CI: 27.2–39.2) for tumors lacking these biomarkers (*P* = 0.005, Fig. 5f).

### Tumor flare is an immune reaction to therapy

**Fig. 6:**
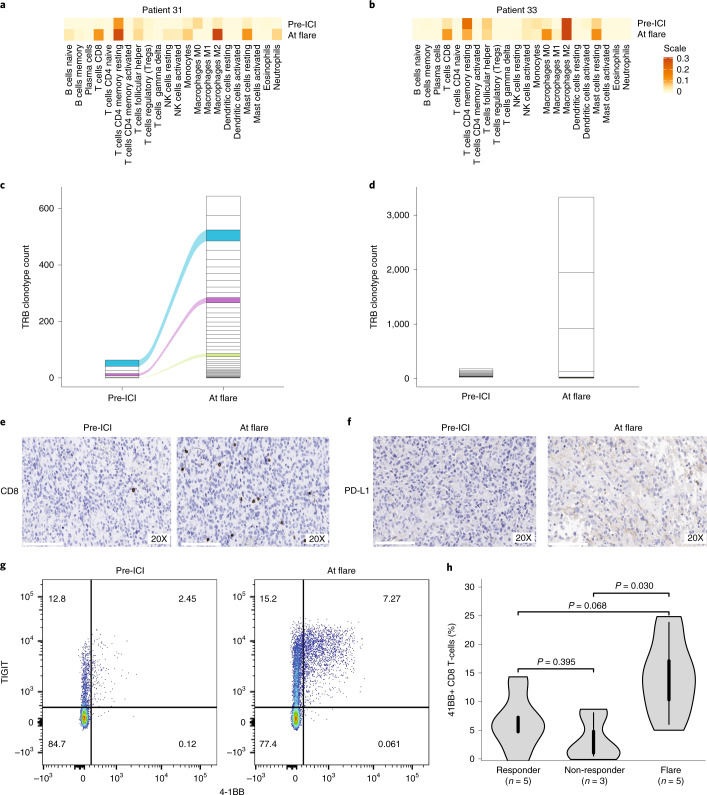
Characterization of the tumor flare response. **a**-**d**, Analysis of 2 patients who had tumor-debulking prior to therapy and at the time of flare. (**a, b**) Total immune cell content in pre-therapy and at flare. **c**,**d**, The corresponding CapTCR-sequencing and T cell receptor clonotype (TCR) analysis in these samples. Each box represents a specific TCR clonotype. Specific clonotypes which were shared between the baseline samples and at flare were tracked using the same colour between the baseline and flare samples. **e**,**f**, Immunohistochemistry for PD-L1 expression, and CD8-T cell infiltration in the pre-therapy sample and at flare, as shown in the representative 20X images from the tumor sample in patient-1 (P33). All immunohistochemistry was analysed by two independent pathologists. **g**, Representative flow cytometry plot showing activation of CD + T cell (TIGIT and 4-1BB) from the blood sample of a patient before treatment initiation and at flare. **h**, 4-1BB + CD8 + T cells in blood from responders without flare, non-responders and flare. For the box-plots, data are represented as median + /- interquartile range. For statistical significance in pairwise comparison of responders, non-responders and flare, the Wilcoxon-Mann-Whitney test was used. All p values are 2-sided.

**Extended Data Fig. 8:**
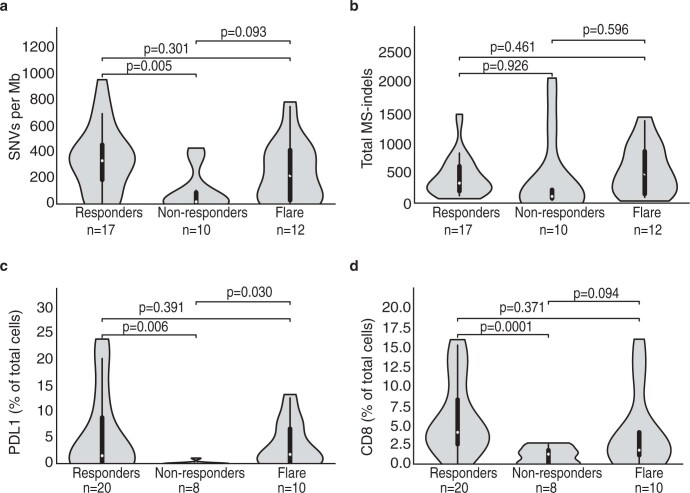
Genomic and immune markers of response in tumours exhibiting flare. Responders without flare, non-responders and stratified by **(a)** SNVs/mb, **(b)** MS-indels, **(c)** PD-L1 and **(d)** CD8 expression. For all box-plots, data are represented as median +/- interquartile range. For statistical significance in comparing responders and non-responders, the Wilcoxon-Mann-Whitney test was used. All p values are 2-sided.

**Extended Data Fig. 9:**
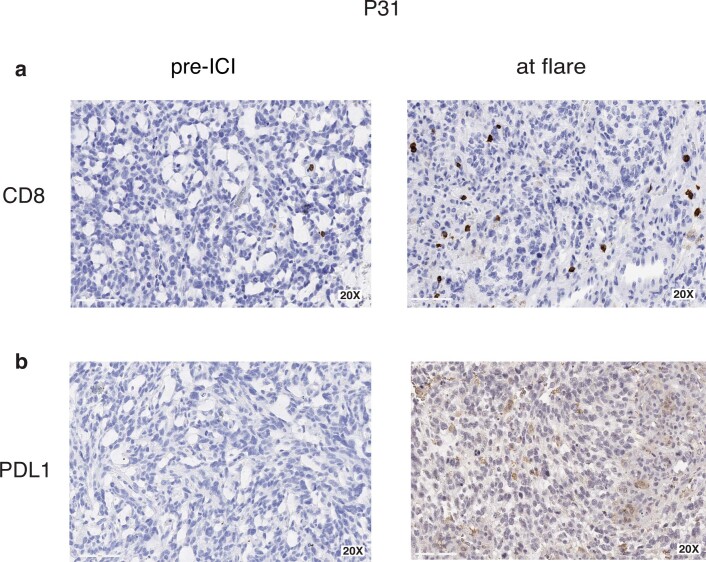
Immunohistochemistry at baseline and flare. Representative images (20X) from patient P31 demonstrating (a) CD8 and (b) PD-L1 expression in pre-ICI and flare samples. All immunohistochemistry was analysed by two independent pathologists.

To determine etiology of tumor flare, we first analyzed the genomic and immune markers of these tumors. Cancers developing flare had pre-treatment genomic and immune characteristics similar to the responders without flare (Extended Data Fig. 8a–d). We then compared the pre- and on-therapy tumors in two patients who had further surgical debulking at flare. Transcriptomic analysis and immune inference using single state deconvolution (Methods) revealed an increase in the overall immune cell expression at flare. Notably, transcriptome signaling revealed that activated CD8+ T cells were significantly increased in both samples following ICI (Fig. 6a,b and Supplementary Fig. 8). Using T cell receptor clonotype analysis (Fig. 6c,d and Supplementary Fig. 9), we observed a dramatic increase of T cell repertoire at flare as compared to their baseline. One sample demonstrated an increase in both clonality and diversity of the T cell population, with some original clones expanding during flare (Fig. 6c), whereas for the other sample, there was reduction in diversity but significantly heightened clonality in the T cell population, involving unique clonotypes harbouring complementary-determining regions (CDR3) not previously reported in public databases (Fig. 6d and Supplementary Fig. 9c). Additionally, increased CD8+ T cell infiltration and PD-L1 expression were observed in both tumors during flare when compared to their pre-treatment samples (Fig. 6e,f and Extended Data Fig. 9). These observations suggest a pre-existing (specific) immune response and further ICI-driven (non-specific and specific) tumor-directed immune expansion at flare.

**Extended Data Fig. 10:**
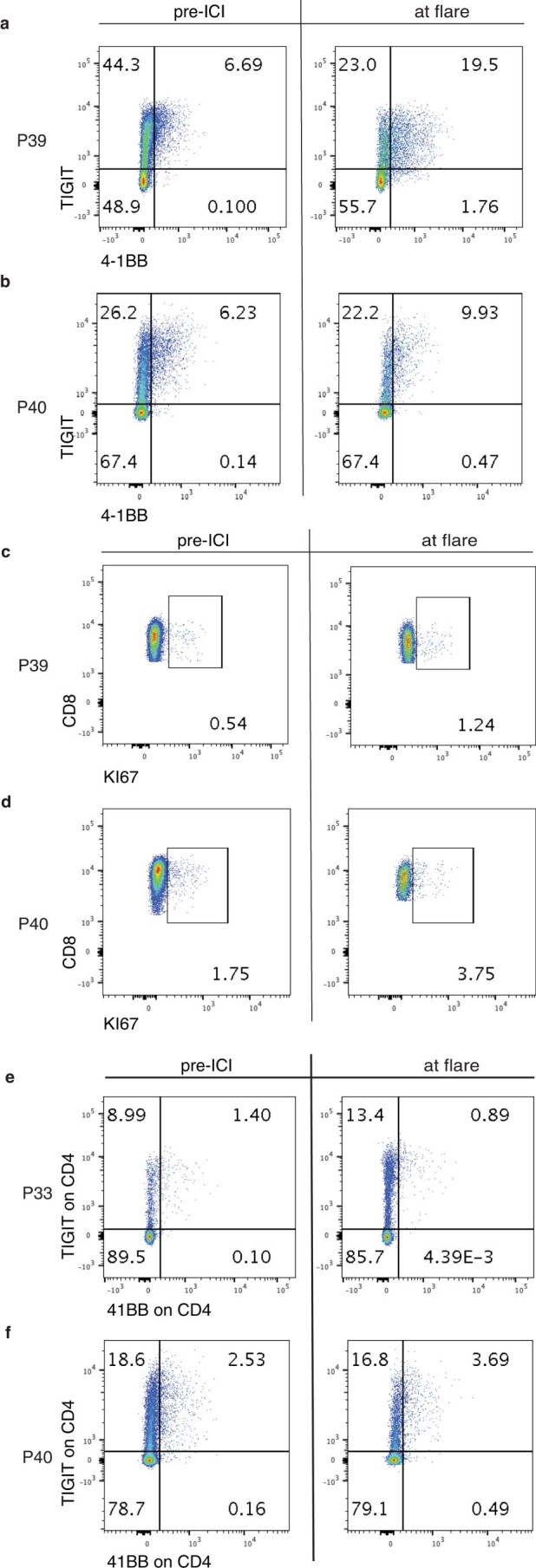
Immune activation in patients experiencing tumour flare. Flow cytometry dot-plots from two patients comparing pre-ICI and flare samples, showing (a, b) TIGIT and 4-1BB expressing CD8+ T-cells, (c, d) Ki67 expressing CD8+ T-cells, and (e, f) TIGIT and 4-1BB expressing CD4+ T-cells.

We next investigated whether this immune activation could be observed systemically (Fig. 6h, Extended Data Fig. 10 and Supplementary Fig. 10). We performed serial flow cytometry analysis of blood samples from multiple patients, prior to treatment initiation and within the first 90 days of initiation of ICI (Methods). Flare was associated with an expansion of peripheral CD8+ T cells expressing Ki67 (a marker of proliferation) and 4-1BB (a member of the TNF receptor family) (Extended Data Fig. 10a–d and Fig. 6g). Detection of Ki67+ CD8+ T cells has been previously reported to be associated with superior response to PD-1 blockade^38^. 4-1BB is well-known as a marker of T cell activation and has co-stimulatory activity for activated T cells^39^. Flare was associated with higher proportion of 4-1BB+ CD8+ T cells in the peripheral blood as compared to non-responders, as well as responders without flare (Fig. 6h). Studies have previously shown that 4-1BB+ CD8+ T cells correlate with response to PD-1 blockade^40^. This uniform increases in 4-1BB and Ki67+CD8+ T cells, was accompanied by an insignificant change in the 4-1BB+ CD4+ T cell population (Extended Data Fig. 10e,f) and no difference between responder, none-responders and flare for the latter (Supplementary Fig. 10a), supporting our hypothesis that CD8+ T cells expressing 4-1BB reflect the expansion of a tumor-specific response. Taken together, our data suggests that flare following PD-1 blockade is with a result of inflammatory response and proliferation of tumor-antigen reactive T cells.

## Discussion

Our study reveals dramatic responses to PD-1 blockade and an associated improved survival for relapsed/refractory hypermutant cancers in children and young adults with germline DNA replication repair deficiency. Several insights can be derived from the sustained responses in different tumor types, and the contributions of SNVs, MS-indels and the microenvironment to both response and flare.

The unique responses to ICIs in children with germline replication repair deficiency are different from previous observations by several groups. First, our data starkly contrast with the lack of ICI efficacy in childhood cancers in general^15,23–25^, and specifically in progressive paediatric brain tumors^41^. The lack of response in paediatric cancers, which is independent of PD-L1 expression^15^ or systemic immune activation^15,24^, has been attributed to the low tumor mutation burden^2,8,27^, low expression of major histocompatibility complex^42^, and the predominance of macrophages in the tumor micro-environment^43^. Some of these causes may need to be re-examined in view of our data.

Second, more than half of replication repair-deficient pediatric CNS tumors had objective responses, resulting in a median survival of 2 years when ICI was continued. This is remarkable, as historically, rapid progression with a median post-relapse survival of merely 2.6 months has been reported in children with replication repair-deficient high-grade glioma^7,27^. Furthermore, these responses contrast with the poor response to ICIs in adult hypermutant gliomas^32,44^. Indeed, comparative mutational analysis in these two cohorts revealed significantly higher mutation burden in paediatric MMRD + PPD gliomas, and higher MS-indels in all germline replication repair-deficient gliomas, as compared to adult hypermutant gliomas (Supplementary Fig. 6). Moreover, ICI-responders among germline replication repair-deficient gliomas exhibited predominance of clonal mutations (Extended Data Fig. 2), which is different from treatment-related secondary MMR deficient adult gliomas, where late acquisition of sub-clonal mutations during tumorigenesis results in suboptimal responses to ICI^32^. Additional causes may include an immunosuppressed tumor microenvironment dominated by myeloid infiltrates^45^ which are frequent in adult gliomas as compared to replication repair-deficient gliomas, where myeloid infiltrates are less predominant (Fig. 5).

Third, all patients who continued ICI therapy for non-CNS solid tumors including disseminated cancers, responded, and are alive at a median follow-up of 2.6 years, with an estimated survival rate of 80%. Although still preliminary, these data are encouraging, as the survival in most recurrent childhood cancers is poor, especially for metastatic disease^46,47^. Lack of response to ICI in previous clinical trials in pediatric recurrent/refractory solid tumors probably reflects the lack of inclusion of DNA replication repair-deficient tumors in these studies^15,23–25^. As we observed responses in metastatic colorectal and genitourinary cancers, our results compare favourably with the otherwise grim survival for such tumors in both children^48,49^ and adults^50,51^ when using conventional therapies. Interestingly, our data compare favourably even with studies using ICI in MMRD cancers in older adults (median age: 60 years, versus 12.3 years in our study), in whom late failures were noted, resulting in 50–55% survival at 2 years^17,18,52^.

Collectively, the dramatic responses and favourable outcome observed in childhood replication repair-deficient cancers can be explained by several key biological features. First, the earlier onset^8^, combined with significantly higher mutations^2^, MS-indels^3^, and neoantigen burden drive CD8+ T cell activation, which is especially robust in children and adolescents when compared to older patients^53^. Second, the additional loss of the polymerase-proofreading mechanism confers genomic mutational signatures (both MS-signatures^3^ and COSMIC signatures^31^ 10 and 14) which may play unique immunogenic role in determining response and survival. Third, given that MMRD + PPD cancers cannot repair errors during DNA replication, the exceptionally high rate of obligatory and continuous accumulation of mutations in these cancers^8^ probably confers ongoing immunogenicity, contributing to immune-surveillance leading to both the durable and the delayed responses observed in our cohort. This probably contributed to the difference between progression-free (post-ICI) and overall survival for patients who continued anti-PD1 treatment after a second progression while on ICI therapy (Fig. 2d and Supplementary Fig. 1b,c). The use of combinatorial therapies targeting additional immune-checkpoints and pathways can therefore be of further benefit for patients whose tumors escape immune surveillance following single-agent anti-PD1 blockade^54^.

An additional observation worth discussion is the response in a tissue-agnostic manner in a patient with synchronous tumors exhibiting favourable genomic and immune biomarkers for response (P01). As cancer immune surveillance is different than the irreversible resistance which occurs upon progression after chemo-radiation approaches, these data support the exploration of neoadjuvant, maintenance, and combinatorial uses of ICI in these patients, to limit toxicities and improve effectiveness of first-line strategies^55^.

There is increasing evidence that responses to ICI cannot be predicted by single biomarker^56^. Our data confirm that this is true in replication repair-deficient cancers which are driven by dysfunction in both SNV and MS-indel repair. Although initially TMB was thought to be the sole contributor to immune response in hypermutant cancers, indels and MS-indels have been suggested to be the important drivers of response to ICI in MMR-deficient cancers^3,35,57^. We add a new dimension to this concept by revealing that in cancers driven by MMRD-only, TMB is relatively lower, and MS-indels drive response, while in MMRD + PPD cancers, the role of MS-indels is attenuated, and TMB is the major driver of response. The dual roles of both mutational mechanisms also affect the microenvironment, with upregulation of PD-L1 and infiltration of CD8+ T cells. Importantly, the combination of both genomic mechanisms and immune markers are powerful predictors of survival in replication repair deficient cancers and should be incorporated as combined biomarkers in future clinical trials. Additionally, the contribution of an immune microenvironment to the ICI response may be relevant in the context of similar findings reported in other subsets of paediatric cancers not known to be driven by replication repair deficiency^58^.

Paradoxically, this hyperactivation of the immune microenvironment can also be detrimental, since tumor flare, which indicates both specific (pre-existing) and non-specific (new) expansion of TCR clones, can be misinterpreted as tumor progression and lead to premature treatment abandonment. In this study, we observed flare to be quite common in germline replication repair-deficient cancers, which are highly immunogenic due to their extreme SNV and MS-indel burden. These flare responses are distinct from hypermutant adult glioblastomas originating as a result of previous chemo-radiotherapy, where true progression is reported more frequently than pseudo-progression^32^. The higher prevalence of flare (26%) in our study, and the prolonged survival in patients who continued therapy, further support this notion. Although vaccine-based approaches lead to a significant local immune response^59^, these may be driven by different mechanisms. Indeed the timing of flare was earlier (median: 34 days) as compared to that reported in studies in adult patients with glioblastoma treated using immune-directed approaches (median: 10 weeks; commonly <3 to up to 6 months)^59^. Although the observations of intra-tumoral inflammatory response at flare are limited and need to be ratified in larger cohorts, our data, which demonstrate similar genomic and immune profile among tumors which responded and those that developed flare, can explain the late responses observed in patients where therapy was continued. This highlights the importance of developing novel functional imaging techniques^60^ and minimally invasive biomarkers^61^ to better predict and diagnose tumor flare, and innovative strategies to modulate this aggressive immune response.

Despite the limitations of a registry study, this is the first description of a large cohort of children and young adults with previously fatal, recurrent/progressive germline DNA replication repair-deficient cancers, demonstrating impressive responses to PD-1 blockade. Importantly, this registry study was able to collect germline and tumor tissue from all patients, including longitudinal blood samples and relapsed tumor tissues whenever repeat surgeries were performed, which has historically not been performed in prospective paediatric studies including recent immunotherapy trials. The robustness and consistency of our results for response, survival and predictive biomarkers despite the heterogeneity of our cohort support a strong biologic rationale for use of ICI in this rare patient population. Although longer follow-up is required to determine whether immunotherapy can be a curative strategy for RRD cancers, the sustained responses and lack of late relapses in a significant number of patients in this cohort are encouraging. This study also sheds light on the complex interplay between the tumor genomic status, microenvironment, and the systemic immune response, especially in the context of extreme mutation and MS-indel burdens. Future trials should prospectively analyse the roles of germline versus somatic deficiency, and the components of the replication repair machinery, to identify patients who are likely to derive maximal benefit from anti-PD-1 immunotherapy. Last, our study highlights the impact of studying a genetic cancer syndrome to understand general cancer processes and deriving direct therapeutic implications for patients.

## Methods

### Study design and patients

Patients were identified through the International Replication Repair Deficiency Consortium (IRRDC), based at SickKids, Toronto. The IRRDC has enrolled >200 patients from 45 countries since 2007. Patients with confirmed /suspected replication repair deficiency were eligible. The SickKids Research Ethics Board approved the study. Consent was obtained from patients and families. This included submission of clinical and imaging data, and tissue and blood samples for centralized analysis. Germline diagnosis of constitutional MMRD, Lynch or PPD were confirmed by the IRRDC’s genetic counsellor (MA), based on the family history, next-generation panel sequencing of germline samples for MMR and *POLE/ POLD1* genes (performed locally or centrally at CLIA-approved laboratories), and immunohistochemical (IHC) staining pattern of the tumor and normal tissues (CH). Thirty-eight patients with 45 cancers who had received treatment with anti-PD1-directed immune checkpoint inhibitor (ICI) therapy are reported here. Patients followed treatment as per the guidelines shared with the collaborators by the IRRDC. These guidelines were derived from ongoing clinical trials, with standard guidelines for monitoring safety and stopping rules for toxicity, as per the ongoing multi-centric clinical trial protocol (NCT02992964). However, patients were not treated prospectively as part of the trial. Ultimately, the choice of the agent and the nuances of therapy remained at the discretion of the treating team. Monthly, and as-needed meetings were coordinated to track progress, address safety concerns if any, and collect data in real-time. Blood samples for companion biomarker studies were collected prospectively before and on therapy following informed consent at specified time-points as per NCT02992964.

Clinical records were reviewed to obtain patient demographics, cancer diagnosis, date of initiation and completion of ICI, choice of ICI agent, and survival outcomes (including date of disease progression and/or patient death). Centralized pathology, radiology, molecular, and biomarker analyses were performed for all. Imaging at baseline and following initiation of ICI were obtained for central review of objective tumor response. For the remaining cases, response (or otherwise) was determined by the assessment of the local treating team. Available scans were centrally reviewed by a radiologist blind to the clinically determined response, and tumor measurements were documented according to the RANO criteria^28^. In brief, the best tumor response was determined as the percentage change in the product of bi-perpendicular dimensions from baseline on the contrast-enhanced T1 images. Complete response (CR) was defined as the disappearance of all enhancing disease (measurable and non-measurable) sustained for at least 4 weeks, with stable or improved non-enhancing FLAIR/T2W lesions, no new lesions and clinical stability. Partial response (PR) was defined as 50% or more decrease of all measurable enhancing lesions sustained for at least 4 weeks, with no progression of non-measurable disease, stable or improved non-enhancing FLAIR/T2W lesions, no new lesions and clinical stability. Stable disease (SD) was defined as images that did not qualify for complete response, partial response, or progression, with stable non-enhancing FLAIR/T2W lesions and clinical stability. Progressive disease (PD) was defined as 25% or more increase in enhancing lesions, with increase (significant) in non-enhancing FLAIR/T2W lesions, not attributable to other non-tumor causes any new lesions, and clinical deterioration not attributable to other causes. For patients with non-CNS solid tumors, the revised RECIST (v.1.1) was used^29^. CR was defined as complete disappearance, PR as at least 30% decrease in sum of the diameters, PD as at least 20% increase in the sum of the diameters of the target lesion, and SD as lack of sufficient change to be classified as CR/ PR/ PD. Patients with objective radiological response (CR/PR) and/ or stable disease (SD) were labelled as ‘responders.’ Among those with progressive disease, patients experiencing rapid early clinical and/or radiological deterioration (with > 100% increase in tumor size within 90 days of starting ICI therapy) were defined as demonstrating a ‘flare’ response and were studied in more detail. For patients able to continue ICI, subsequent imaging was reviewed to confirm response or progression. Those with sustained clinical and/or radiological progression despite continuation of ICI treatment were classified as ‘non-responders.’ For biomarker prediction analyses, ‘responders’ also included those with an initial ‘flare,’ who continued on ICI and demonstrated delayed responses.

### Whole exome sequencing and analysis

Genomic DNA from 39 tumors, along with matched germline blood samples, was extracted using the PaxGene Blood DNA Extraction Kit (Cat No./ID: 761133) for blood samples, Qiagen DNeasy Blood & Tissue Kits (Cat No./ID: 69504) for frozen tissue, MasterPure Complete DNA and RNA Purification Kit (Epicentre #MC85200) for paraffin embedded tissue). WES was performed at The Centre for Applied Genomics (TCAG), SickKids, using SureSelect Agilent All Exon v5 kit, followed by sequencing (100X) on Illumina HiSeq 2500. The software bcl2fastq2 v2.17 was used to generate raw fastq files. Alignment to the hg38 reference genome using Burrows-Wheeler Alignment version 0.7.12 (ref. ^62^), followed by pre-processing which included flagging PCR duplicate reads using Picard MarkDuplicates tool (version 1.130). For each tumor and normal tissue data pair, regions with insertions and deletions (Indels) were realigned using GATK IndelRealigner (version 1.130) to minimize number of mismatched bases across all reads, followed by base recalibration. This was adapted from the GATK best practices for whole exome and genome analysis^63–65^ optimized for our laboratory’s workflow^66^. Somatic variant calling was done post-alignment, using processed bam files from tumor and matched normal samples, to call both single nucleotide variants (SNVs) and insertion deletion (indel) variants. A consensus vcf file of shared variants across 2 or more of 4 variant callers (Mutect v1.1.5)^67^, GATK v3.6/Mutect2, Strelka v1.0.14 (ref. ^68^), and Varscan2 Somatic v2.4.2 (ref. ^69^) was generated for SNVs and indels separately, using VCFtools v.0.1.15 (ref. ^70^), and these vcfs were annotated using VEP v83 (ref. ^71^). The TMB from WES data was calculated by counting total number of somatic SNVs divided by total number of callable bases in megabases (~50 Mb). DeconstructSigs^72^ was used to determine COSMIC signatures^31^ in the mutation spectrum within a tri-nucleotide context for each sample. All bioinformatics analyses were performed on the SickKids and UHN High-Performance Clusters.

### HLA-typing

Paired end fastq files from matched germline WES data were used as input to computationally determine HLA Class-I types for 39 tumors using a consensus of HLAminer^73^, and HLAVBSeq^74^, as described and validated in a previous study in our laboratory^75^. The top 6 HLA-types were used as input for neoantigen calling as described below.

### Neoantigen calling

The Mutect2 vcfs generated for each tumor (described above) were used as input along with bioinformatically generated HLA-types (above) for MuPeXI^76^, to get a list of strong binding candidate neoantigens per HLA-type. This uses netMHCpan^77^ (to calculate all variant peptides ranging from 8–12 mer, and total candidate neoantigens was determined by selecting all neoantigens that showed ‘%rank’<0.5 binding affinity, denoting all strong binders (as recommended by the netMHCpan user manual). It is to be noted that all candidate neoantigens were restricted to class-I MHC proteins only.

### Microsatellite indel calling

Microsatellite indels were called on the bam files of tumor and matched normal samples, using an in-house pipeline using MSMuTect v1.0 (ref. ^36^). The detailed methods for this algorithm have been previously reported^36^. In brief, repeats of five or more nucleotides were considered to be MS loci, and using the PHOBOS algorithm and the lobSTR approach, tumor and normal BAM files were aligned with their 5’ and 3’ flanking sequences. Each MS-locus allele was estimated using the empirical noise model, which is the probability of observing a read with a microsatellite (MS) length k and motif m, where the true length of the allele is j with the motif m. This was used to call the MS alleles with the highest likelihood of being the true allele at each MS-locus. The MS alleles of each tumor and matched normal pair were called individually, which were compared to identify the mutations on the tumor MS-loci. The Akaike Information Criterion (AIC) score was assigned to both the tumor and normal models, and a threshold score that was determined using simulated data was applied to make the final MS-indel call.

### Neoantigens from coding microsatellites

Microsatellite indels were called using MSMuTect v1.0 (ref. ^36^). The Indels were annotated using the Ensembl Variant Effect Predictor^71^ (VEP release/104.2). Neoantigens were identified using pVAC-seq^78^ software suite. Using NetMHCpan4.1 (ref. ^77^) algorithm (included in pVAC-seq), we predicted 8- and 9-mer neoantigens with strong binding affinity (score ≤ 500 nM) to the patients’ HLA class I (A, B, or C).

### Clonal TMB analysis

To determine the clonal status of each mutation, we first determined allele-specific tumor copy number by applying FACETSv0.5.6 to the 39 tumor-normal exome pairs^79^. The snp-pileup command was run using the parameters –q15 –Q20 –P100 –r25,0 to generate the necessary allele fraction and depth information for FACETS. FACETS was then run using default parameters. To ensure accuracy, manual review of the FACETS copy number estimate solutions was conducted for each of the 39 tumor-normal pairs. Many hypermutant tumor samples are typically diploid, which can confound copy number estimation. In these cases, we manually assigned the tumor’s diploid status (based on LogR and BAF plots showing no copy changes), and manually assigned the purity by doubling the value of the highest variant allele fraction peak (as would be expected in a diploid tumor with a mutation on one tumor allele). A custom script was then used to merge the total and alternate read counts for each mutation with the sample purity and allele-specific copy number estimates for input into the ABSOLUTE method. We used the ABSOLUTE method to determine the cancer cell fraction, clonal or sub-clonal status of each SSM mutation, and confidence intervals of the cancer cell fraction estimate^80^. The ABSOLUTE method functionally achieves the same aim of mutation clonality status assignment as PyClone. Mutations were defined as clonal if the 95% confidence interval of the cancer cell fraction estimate overlapped with 1 and sub-clonal otherwise. For a number of tumors, reliable copy number, mutation and purity estimations could not be extracted, rendering clonal architecture analysis intractable and these tumors were omitted from the analysis. In the end, clonal and sub-clonal SNVs were computed and are reported for 21 tumors.

### Immunohistochemistry

Four-micron thick sections of formalin-fixed paraffin-embedded (FFPE) surgical specimens were stained using an automated stainer (Dako-OMNIS) with the following primary antibodies: PD-L1 (clone:28-8, Abcam), CD68 (Clone:PG-M1, Dako-OMNIS), CD8 (Clone:c8/144B, Dako-OMINS), CD3 (polyclonal rabbit, Dako-OMNIS), and CD4 (Clone:SP35, Sigma-Aldrich). Quantitative evaluation of the immunohistochemical stains was performed by examining each section using at least 5-7 different high-power fields with the most abundant tumor-infiltrating lymphocyte areas. The tumor was considered PD-L1 positive if ≥1% of tumor cells exhibited a circumferential and/or partial linear plasma membrane PD-L1 staining of tumor cells at any intensity^81^. The percentage of infiltrating immune system cells was estimated by manual eyeballing as none, mild, moderate, and severe (0 = none, <10%=mild, 10–50%=moderate, >50%=severe). For downstream analyses, infiltration higher than the median values of the continuous data of immune infiltrates was used to classify tumors as ‘high’ or ‘low’ infiltration for each marker. All immunohistochemistry was reviewed blindly and independently scored by two teams of pathologists (CH/NA and OK) centrally with good concordance (Supplementary Fig. 7).

### Immune-inference analysis

RNA was extracted as per standard-kit protocol from tumors biopsied at both, baseline and time of flare, in 2 patients (P31, P33) and submitted for total r-RNA depletion RNAsequencing on HiSeq 2500, at TCAG. Following sequencing, 126 bp paired-end reads from the raw data were aligned to the hg19 reference genome generated using STAR aligner v.2.4.2a (ref. ^82^), followed by RSEM v.1.2.21 (ref. ^83^) expression analysis to generate a gene-expression matrix for each sample using the TPM values. The analysis was restricted to only coding genes. This was then run through CIBERSORT in the absolute mode^84^, to generate immune inference data for 22 immune cell subtypes. The immune inference results from CIBERSORT_absolute^85–88^ were plotted using ‘ComplexHeatmap’^89^ package on Rv.3.5. In addition, immune inference was also derived using a complementary deconvolution method EPIC^90^ as previously published and we found concordance between CIBERSORT_absolute and EPIC, which was represented as side-by-side bar-plots (Supplementary Fig. 8). The parameters used for running EPIC involved tumor-signature matrix to call tumor infiltrating cells. These results were then exported as cell fractions.

### T cell receptor rearrangement repertoire (TCR) profiling

Genomic DNA was extracted (above) from tumors biopsied at both baseline and at time of flare in 2 patients (P31 and P33). Library preparation and capTCRseq^91^ hybrid capture were performed. Following library preparation, the samples were sequenced first on a MiSeq for QC purposes and then 300 ng of each sample, pooled in a ratio of 1:1:1, was processed for a 3-step capture using target hybrid capture panel^91^. Post-capture QC was performed on a MiSeq, followed by sequencing of up to a depth of ~2 million reads on the NextSeq. After sequencing, the raw data were analyzed using MiXCR version 2.1.12 (ref. ^92^), ‘iNext’, ‘immunarch’ R packages and Pugh Lab customized functions on R version 3.5 to look at T cell receptor rearrangements in the form of unique clonotypes (VDJ rearranged sequences) for T cell receptors alpha, beta, gamma and delta. As the total read depth varied across the cohort, affecting the total successfully aligned reads, all raw fastq reads were down-sampled to ~1 million reads. QC parameters of percent aligned reads, reads used in clonotypes, final clonotype count and the total number of clonotypes per 1000 reads were considered. To quantitatively explain TCR diversity and clonality, we constructed diversity profiles^93^ of each sample (Supplementary Fig. 9), which is a continuum of hill numbers with varying orders (q = c (0, inf)). Hill numbers were calculated as:

$${}^qH = \left( {\mathop {\sum }\limits_{i = 1}^S p_i^q} \right)^{1/(1 - q)}$$

where S was the number of clonotypes in the assemblage, pi was the relative abundance of the ith clonotype and q was defined as the order of the diversity 1. As q determines the sensitivity to clonotypes’ relative abundances. q = 0 returned the most intuitive and frequently used measure of clonal diversity (Richness), which was the count of the number of clonotypes present in a sample. In order to describe the amount of distinct clonotypes present in a dataset, while taking into account the relative abundance of the clonotypes, we added another popular measure based on q → 1 limit of the above-mentioned (Shannon diversity), calculated as follows:

$$H_{Shannon} \equiv H_1 \equiv \mathop {{\lim }}\limits_{q \to 1} H_q = - \mathop {\sum }\limits_{i = 1}^S p_i\,ln\,p_i$$

The parameter q was used to emphasize or de-emphasize the weight of abundance or rare clonotypes. We kept increasing the q and calculating the diversities. As q increased, all the clonotypes, except for dominant ones, were discounted and the resulting value was interpreted as the effective number of dominant clonotypes in the sample. To overcome the known caveat of TCR-sequencing in detecting false absence of a CDR3 due to tumor heterogeneity or insufficient sequencing, we devised a method to calculate the completeness of sequencing data and ensure comparison of samples of equivalent completeness. This prevented inaccurate comparisons between diversity signatures of different samples. For each sample, we constructed models to calculate the completeness of sequencing data to minimize the false absence events taking place due to sequencing insufficiency. One of the samples in our study reached a mathematical saturation point, where first derivatives of the clonotype count versus total sequencing reads functions reached zero. One of the samples, however, was not sequenced to enough depth to reach saturation. Even though the depth was not enough to fully cover all the clonotypes present within the repertoire, the sample showed higher richness compared to its pre-ICI repertoire, supporting our observation regarding increased richness at time of flare.

### Flow cytometry

Viable frozen peripheral blood mononuclear cells were incubated with Fc block (BD Biosciences) prior to staining for surface markers (anti-CD3-clone UCHT1, anti-CD4–clone RPA-T4, anti-CD8–clone RPA-T8, anti-4-1BB–clone 4B4-1, anti-TIGIT– clone MBSA43, anti-Ki67–clone 20Raj1) and viability dye (eBioscience). Cells were fixed and permeabilized for intercellular staining with the Foxp3 transcription factor staining buffer set (BD). Flow cytometry voltages were set using Rainbow beads (Spherotech) with the same setting between experiments. Samples were acquired on a BD LSR Fortessa flow cytometer and data were analyzed using the FlowJo software.

### Statistical analysis and reproducibility

Overall survival (OS) and event-free survival (EFS) was estimated using Kaplan-Meier statistics and determined from the date of initiation of ICI therapy. Patients without an event were censored at the date of last known contact. Uniquely in this population, several patients had multiple separate synchronous malignancies and therefore in these analyses, survival is presented for each individual cancer/tumor in addition to analyses per patient. For example, for the tumor-wise analysis, a patient experiencing an event related to one cancer diagnosis, was shown as censored (rather than an event) for a second synchronous cancer. Univariable logistic regression, estimated through generalized estimating equations, was fitted to assess association between each clinical factor and response. Specifically, for ethnicity, patients were divided into three groups: Caucasian, Hispanic, and others [including Asians (n=2), African-American (n=1), and aboriginal Australian (n=1) due to individual low numbers]. Fisher’s exact test was used to assess association between tumor site and response (due to zero-count cells). Univariable Cox-regression model with robust standard errors was fitted to assess association between each clinical factor and OS. Log-rank test was used to assess association between tumor site and survival. For all biomarkers used in survival analyses, the median for each analysed cohort was chosen as the cut-off for high versus low. Correlation between biomarkers was tested using Spearman’s rank correlation co-efficient test and effects-sizes were estimated for independent predictor variables. Univariable logistic regression, estimated through generalized estimating equations, was fitted to assess association between each biomarker and response. Univariable Cox-regression model with robust standard errors was fitted to assess association between each biomarker and OS. Multivariate analyses were performed for SNVs and MS-indels. Multivariable logistic regression, estimated through generalized estimating equations, was fitted to assess association of SNVs (cut-off at median) and MSI (cut-off at median) with response. Multivariable Cox-regression model with robust standard errors was fitted to assess association of SNVs (cut-off at median) and MSI (cut-off at median) with overall survival. Statistical significance was calculated using Welch’s unequal variances t-test and the Wilcoxon–Mann-Whitney test, for parametric and non-parametric data, respectively. Among MMRD tumors, a single outlier (annotated with a star) was excluded from p-value estimation, as this was a brain tumor which transformed from low to high-grade over several years which plausibly led to higher MS-indel accumulation. All p-values were 2-sided, with a cut-off of 0.05 for significance. Statistical analyses were performed with SPSS v.20, R v.3.5 and Python v.2.7. The generated plots were edited for aesthetics using Adobe Illustrator v.23.0.1.

### Reporting Summary

Further information on research design is available in the Nature Research Reporting Summary linked to this article.

## Online content

Any methods, additional references, Nature Research reporting summaries, source data, extended data, supplementary information, acknowledgements, peer review information; details of author contributions and competing interests; and statements of data and code availability are available at 10.1038/s41591-021-01581-6.

## Supplementary information

Supplementary Information Supplementary Figs. 1–11 and Supplementary Table 1

Reporting Summary

## Data Availability

All data relevant to this work are available at the European Genome Phenome Archive (EGA: https://ega-archive.org/studies/EGAS00001005579; Study EGAS00001005579; Dataset EGAD00001008036) and can be accessed through communication with the corresponding author. Clinical data are listed in Supplementary Table 1. Reference genomes were downloaded from the publicly available resources at https://genome.ucsc.edu. Source data are provided with this paper.

## Source data

Source Data All Excel with all clinical and sequencing results used for statistical analyses

## Extended data

are available for this paper at 10.1038/s41591-021-01581-6.
