# Supplementary material for: Genomic predictors of response to PD-1 inhibition in children with germline DNA replication repair deficiency
Source: Nat Med. 2022 Jan 6;28(1):125–35. doi: 10.1038/s41591-021-01581-6 (PMC8799468; doi:10.1038/s41591-021-01581-6)
Supplement: Supplementary file 1 — Supplementary Figs. 1–11 and Supplementary Table 1 [file 41591_2021_1581_MOESM1_ESM.pdf]

nature portfolio

https://doi.org/10.1038/s41591-021-01581-6

Supplementary information

Genomic predictors of response to PD-1 inhibition in children with germline DNA replication repair deficiency

In the format provided by the authors and unedited

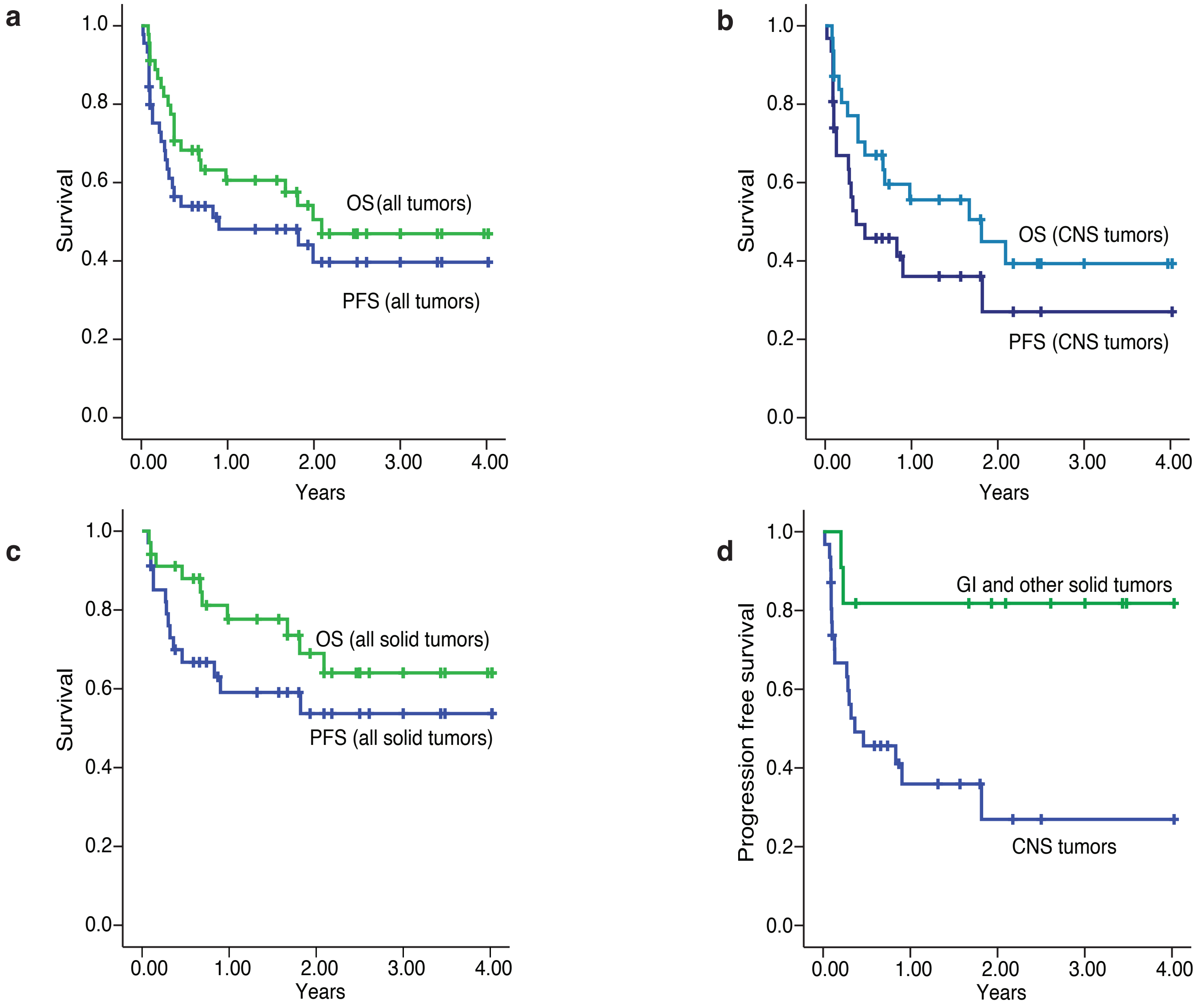

Supplementary Fig.S1. KM estimates of progression-free and overall survival for **(a)** all tumours, (**b**) CNS tumours, **(c)** all solid tumours (CNS and non-CNS). **(d)** Progression free survival for non-CNS solid tumours versus CNS tumours (p=0.01; 2-sided). The log-rank test was used to compare between the groups.

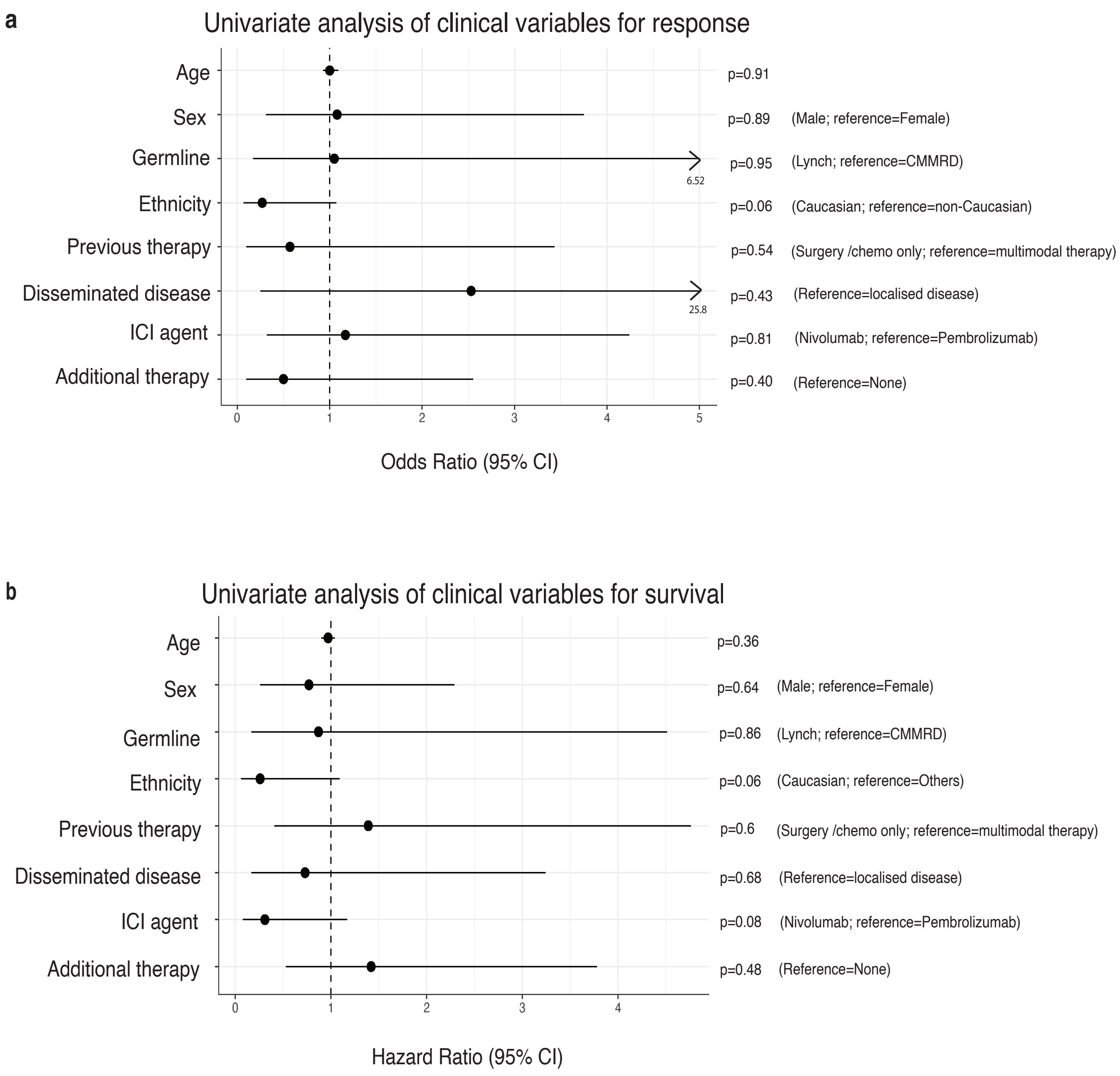

Supplementary Fig.S2. Univariable analysis of clinical predictors for **(a)** response and **(b)** survival following ICI at recurrence for replication-repair deficient cancers. Univariable logistic regression, estimated through generalized estimating equations, was fitted to assess association between each clinical variable and response. Univariable Cox regression model with robust standard errors was fitted to assess association between each clinical variable and survival. All p-values are 2-sided.

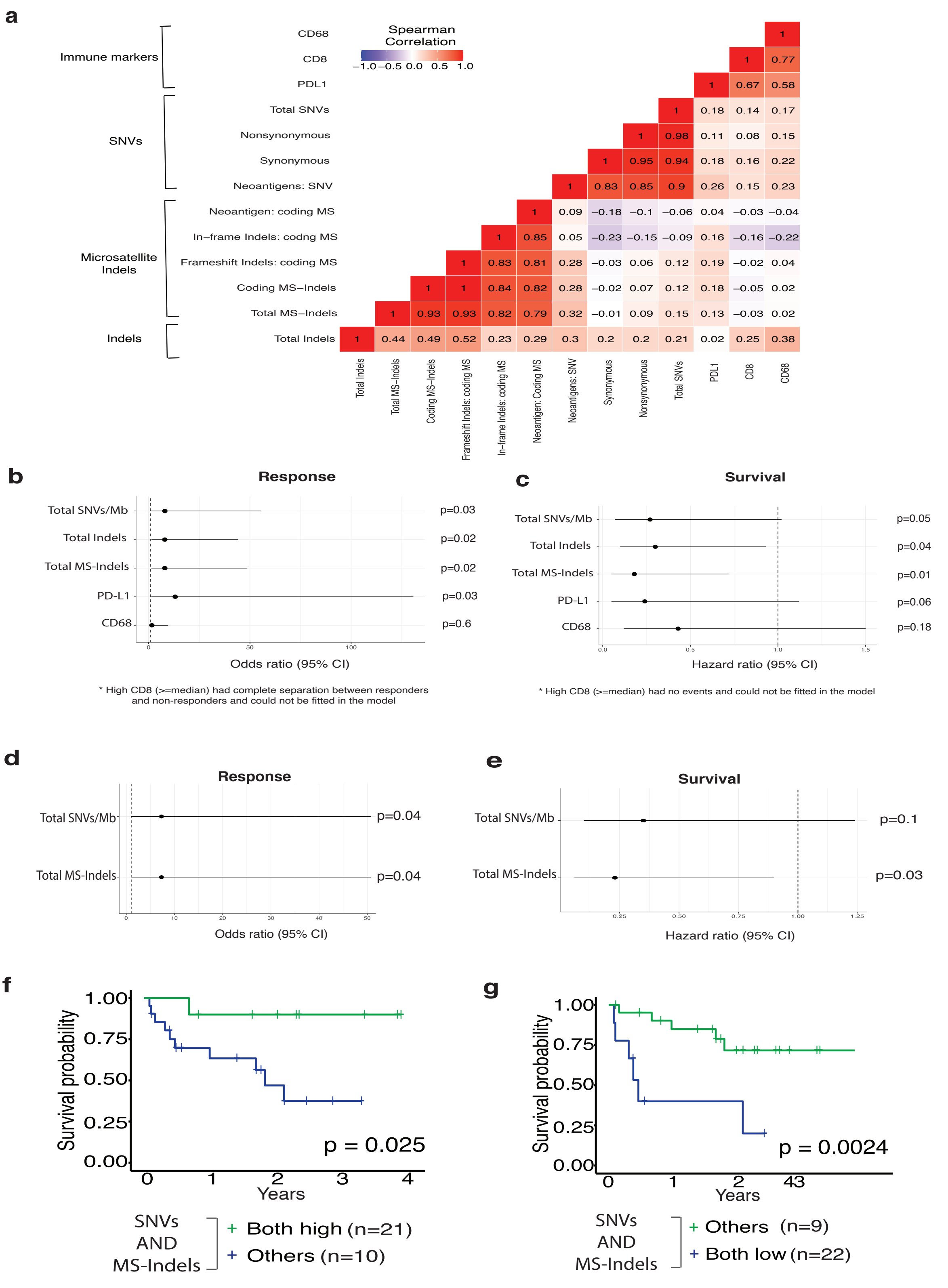

Supplementary Fig.S3. Biomarkers for response to ICI in replication-repair deficient cancers. (a) Spearman's rank correlation coefficient for dependent and independent variables**. (b, c)** Effects size (95% confidence intervals) for univariable analyses for the independent predictors for **(b)** response (assessed using univariable logistic regression) and **(c)** survival (assessed using univariable Cox regression model). **(d-g)** Dual role of SNVs and MS-indels in predicting outcomes: Multivariable analyses for **(d)** response (using logistic regression) and **(e)** survival (using Cox regression model) showing effects size and 95% confidence intervals. Kaplan-Meier (KM) curves for tumors with **(f)** both high and **(g)** both low SNVs and MS-indels (cut offs at the median values). All p-values are 2-sided.

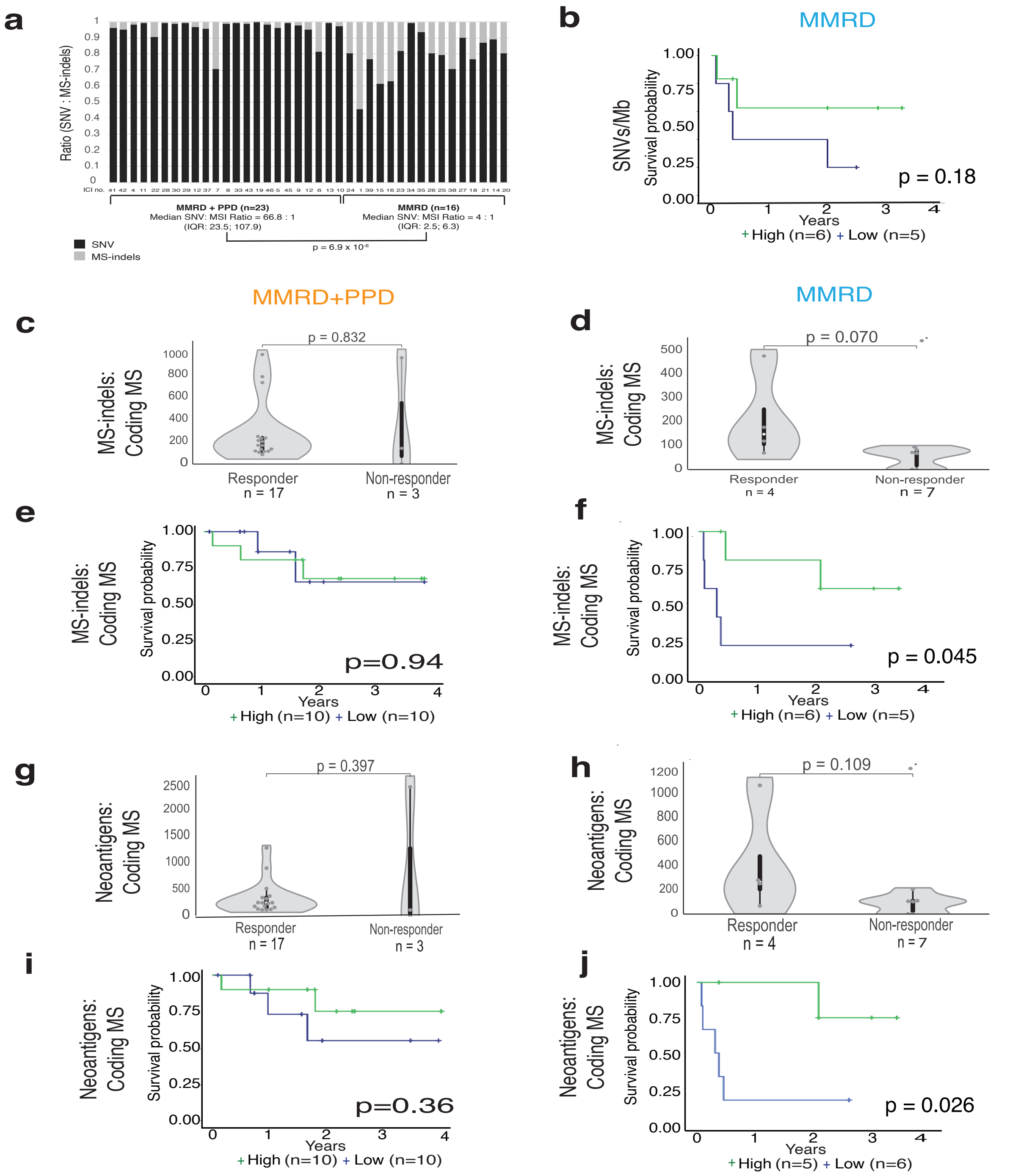

Supplementary Fig.S4. **(a)** SNVs outnumber MS-indels in tumors with MMRD+PPD (Wilcoxon-Mann-Whitney test). Each bar represents the SNV count (black) and MS-indel (grey) for each individual tumor with identifiers mentioned on the x=axis. **(b)** Overall survival for MMRD tumors by SNVs/Mb (>=median). **(c-f)** Response and survival for MS-indels in coding microsatellites in MMRD+PPD versus MMRD tumors. **(g-h)** Response and survival for neoantigens generated by MS-indels in coding microsatellites in MMRD+PPD versus MMRD tumors. For survival, median values were utilized to stratify into 'high' and 'low' groups. For all box-plots for responders and non-responders, data are represented as median +/- interquartile range. For statistical significance in comparing responders and non-responders, the Wilcoxon-Mann-Whitney test was used. Survival analysis was performed using the Kaplan-Meier method, and the log-rank test was used to compare groups. All p values are 2-sided.

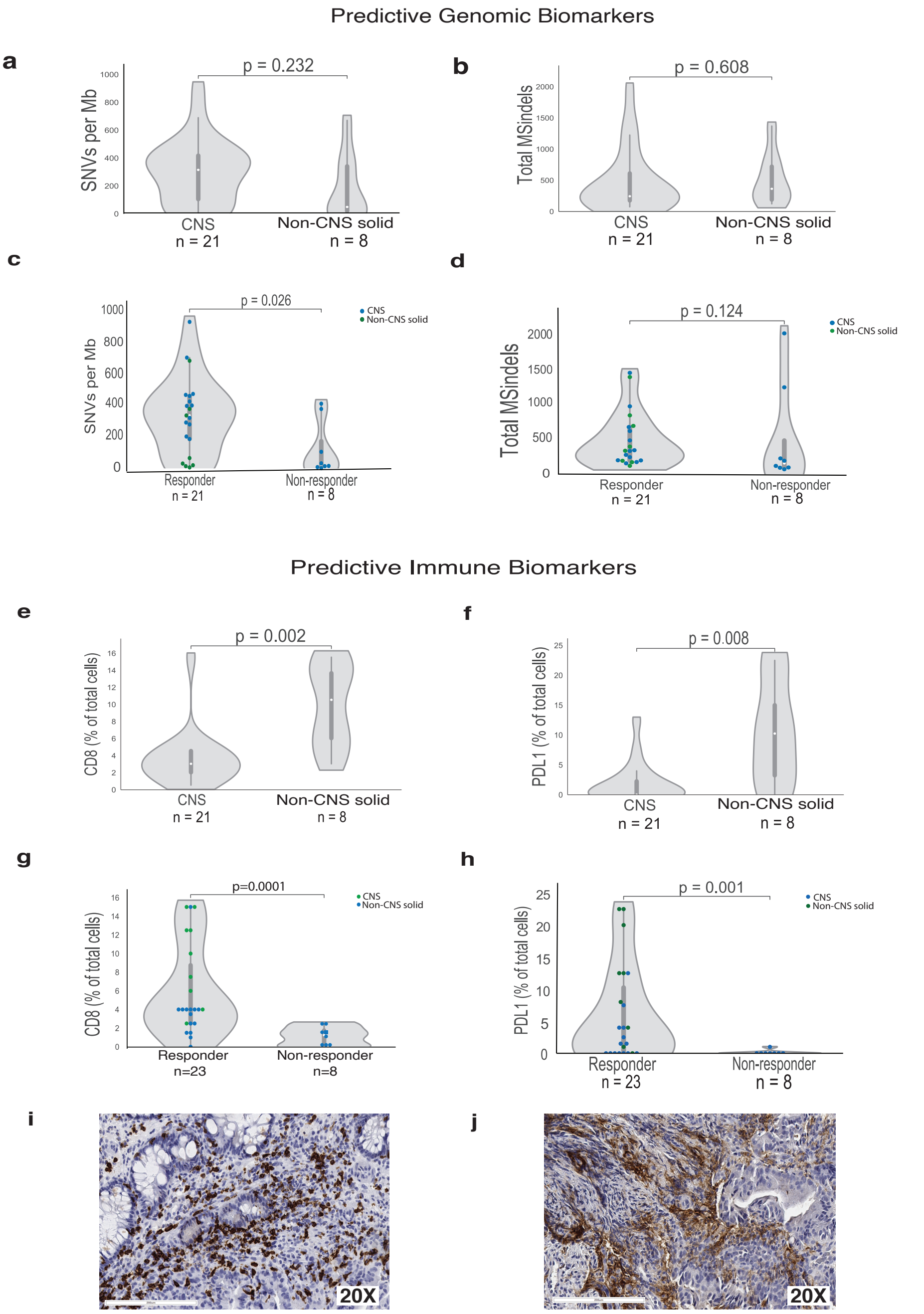

Supplementary Fig.S5. Predictive Genomic and Immune Biomarkers for CNS versus non-CNS solid tumors (excluding haematological malignancies). (a) SNVs and **(b)** MS-indel burden were similar for CNS and non-CNS solid tumors. Responders and non-responders stratified by **(d)** SNV and **(e)** MS-indel burden and tissue type (Green- Non-CNS solid tumors, blue- CNS tumours). **(e)** CD8 and **(f)** PDL1 were significantly higher for non-CNS solid tumors. Responders had higher levels of **(g)** CD8 T-cell infiltration and **(h)** PD-L1 staining as compared to non-responders in all tumours (Green- Non-CNS solid tumors, blue- CNS tumours). Representative images from non-CNS solid tumours, all of which demonstrated **(i)** high CD8 and **(j)** PD-L1 expression (20X). All immunohistochemistry was analysed by two independent pathologists. For all box-plots for responders and non-responders, data are represented as median +/- interquartile range. For statistical significance in comparing responders and non-responders, the Wilcoxon-Mann-Whitney test was used. All p values are 2-sided.

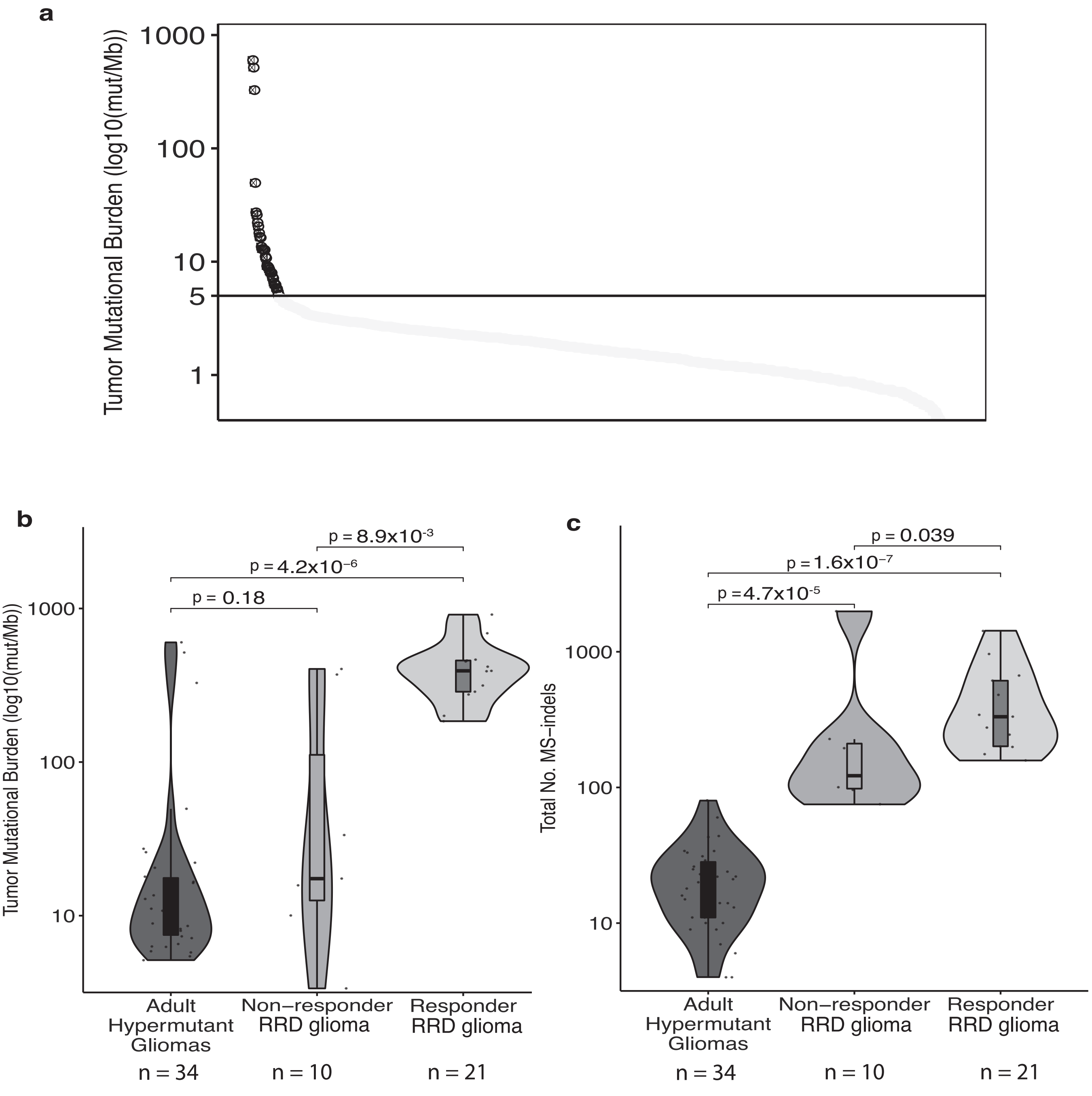

Supplementary Fig.S6. **(A)** TCGA glioma dataset demonstrated 34 tumors (6.8%) with TMB >5 mutations/Mb. **(B)** Extreme mutation burden in replication-repair deficient (RRD) glioma responders (n=21) as compared to non-responders (n=10) and adult hypermutant gliomas (n=34). **(C)** High MS-indel burden in replication-repair deficient (RRD) gliomas (responder: n=21; non-responder: n=10) as compared to adult hypermutant gliomas (n=34). For statistical significance in comparing responders and non-responders, the Wilcoxon-Mann-Whitney test was used. All p values are 2-sided.

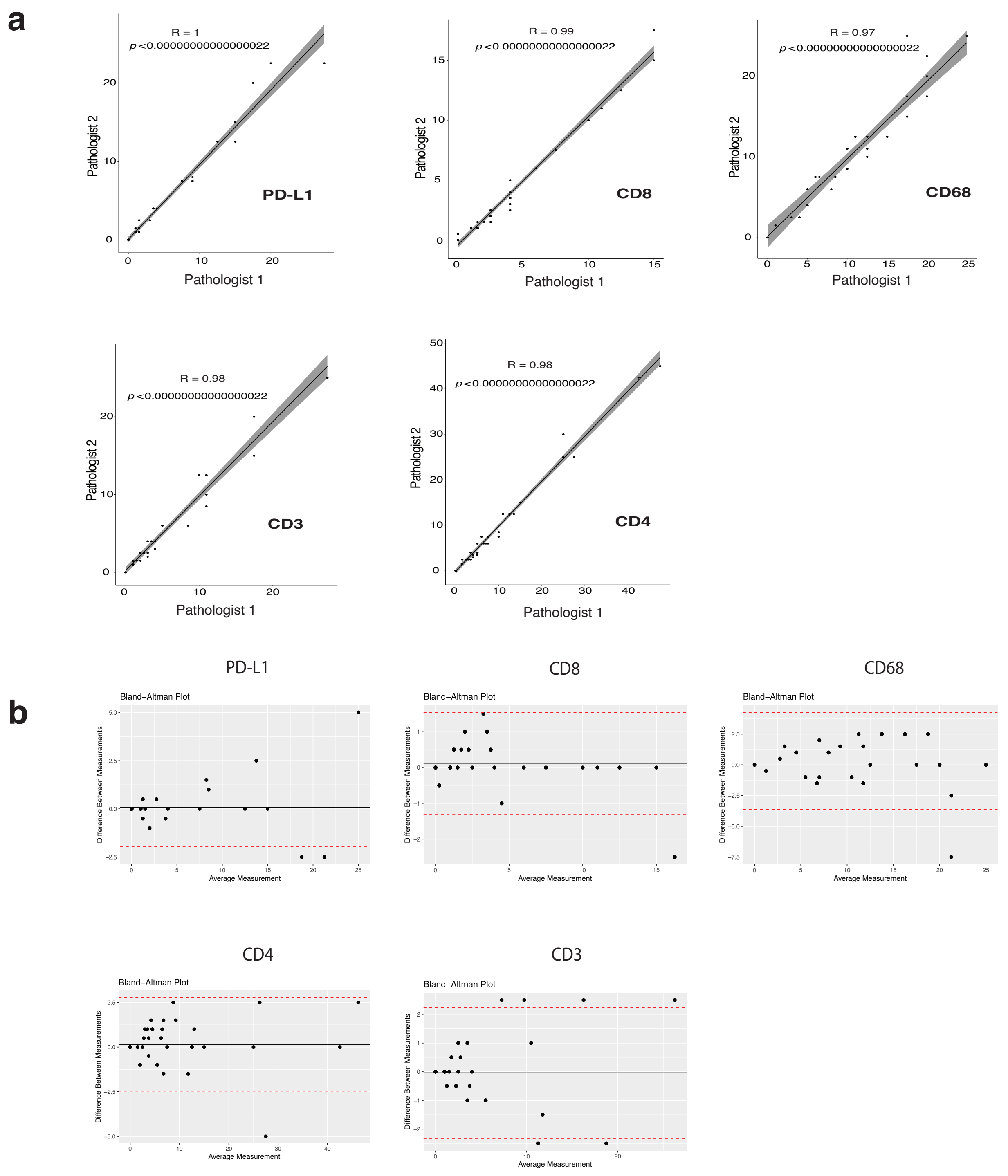

Supplementary Fig.S7. Concordance between immunohistochemical analyses and interpretation between two independent pathologists (CH/NA and OK) shown using **(a)** Spearman's rank correlation and **(b)** Bland-Altman plots.

(Red dotted lines: 95% confidence intervals)

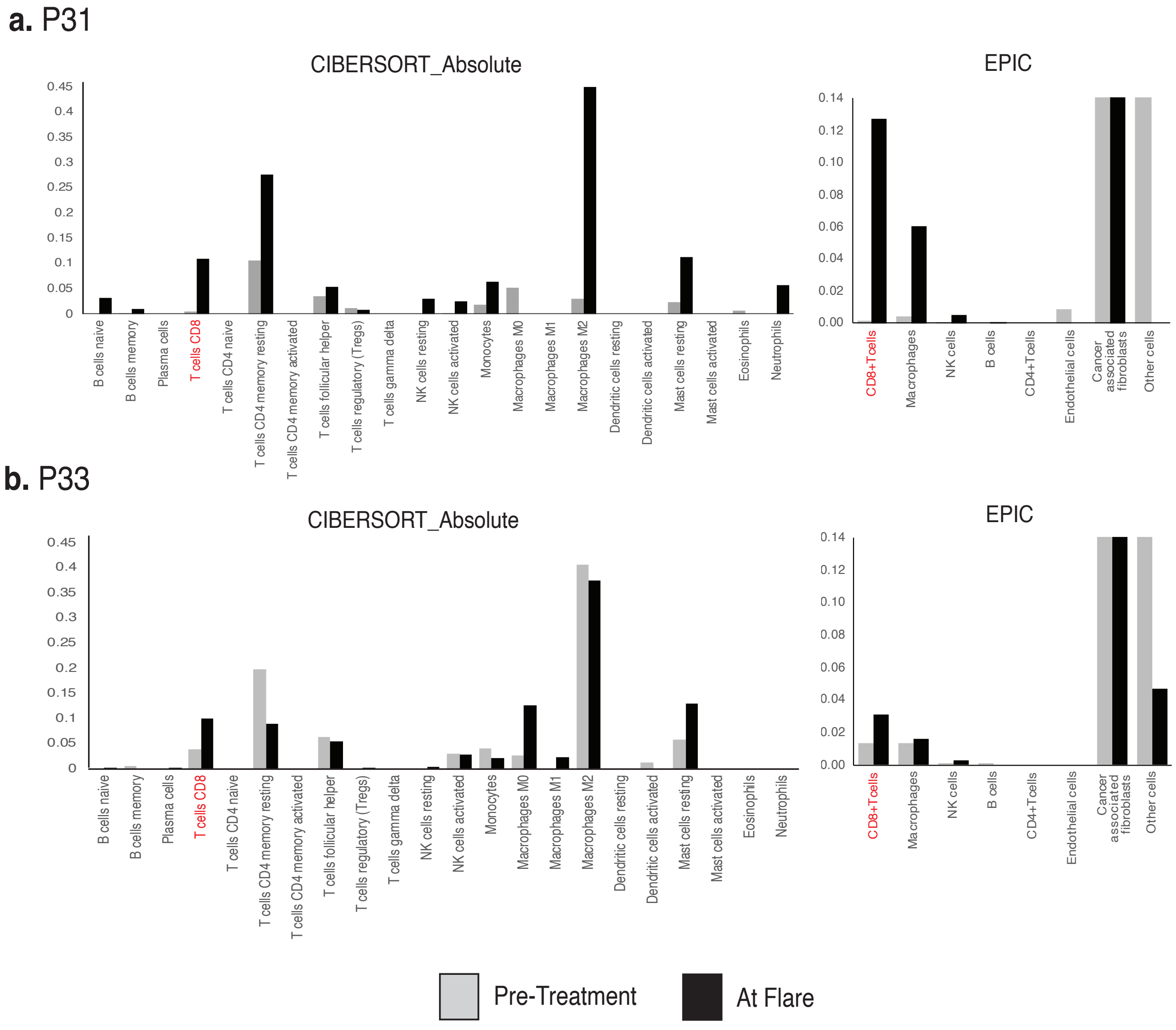

Supplementary Fig.S8. Immune inference of tumor microenvironment at baseline and at flare. Deconvolution analyses of tumor samples of two patients **(a)** P31 and **(b)** P33 using CIBERSORT_Absolute and EPIC (CD8+ T-cells are highlighted in red). **(c)** and **(d)** Same tumors demonstrating the actual abundance of all cell populations.

a

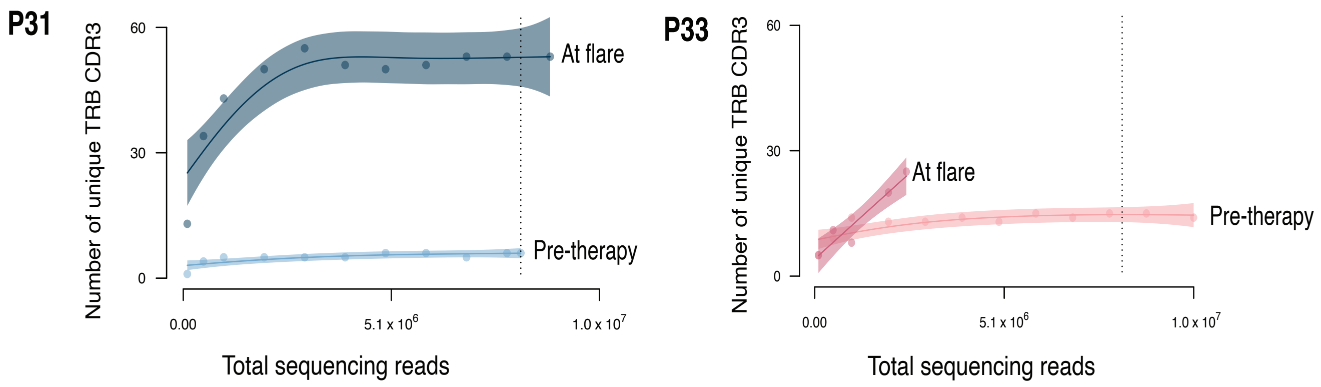

b P31

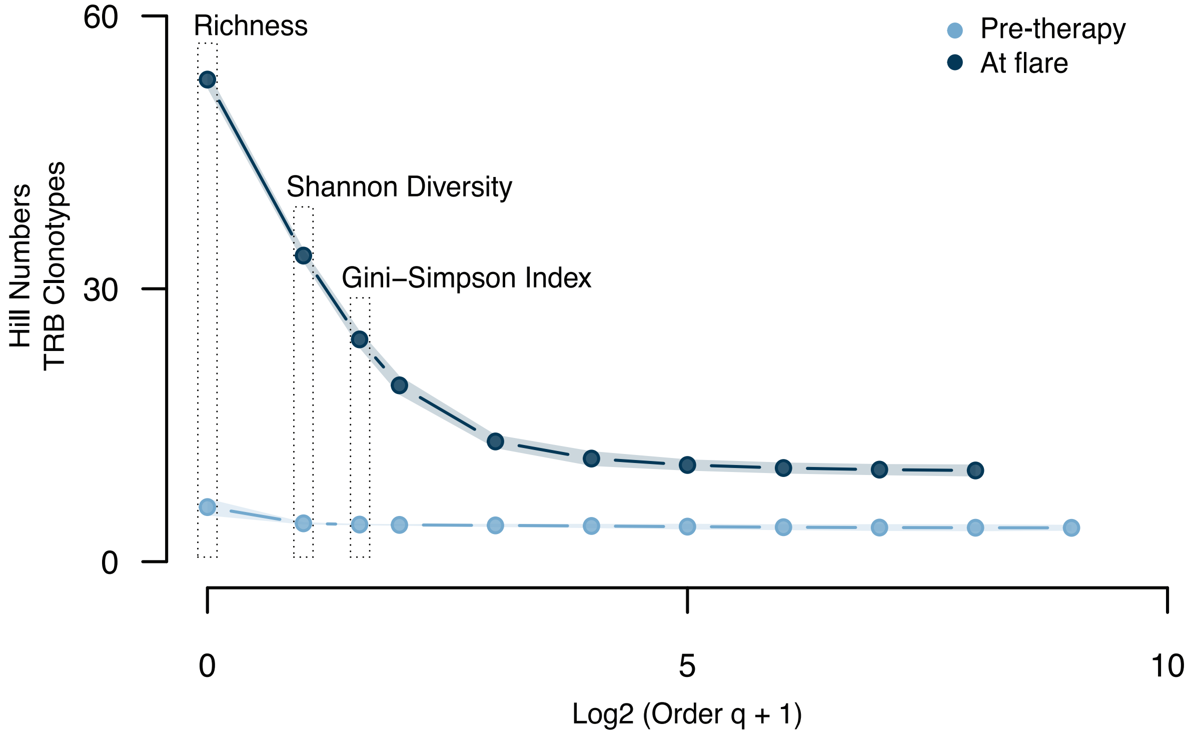

c P33

T-cell Receptor	Clone Count	aaSeqCDR3	IEDB	VDJdb	McPAS-TCR
TRB	1414	CASSQGGWDR_PTAYQETQYF	Not reported	Not reported	Not reported
TRB	1034	CATSVQGYNEQFF	Not reported	Not reported	Not reported
TRB	785	CASSPGGLTTGNTIYF	Not reported	Not reported	Not reported

Supplementary Fig.S9. **(a)** Sequencing reads for two patients at diagnosis and at time of flare (Data are presented as mean values +/- SEM). **(b)** TRB richness and diversity measures at flare compared to baseline for P31 (sequencing had reached saturation for both samples at pre-therapy and at flare). **(c)** Complementary-determining regions (CDR3) sequences of the major three clonotypes detected at flare in P33 (TRB: T cell receptor beta).

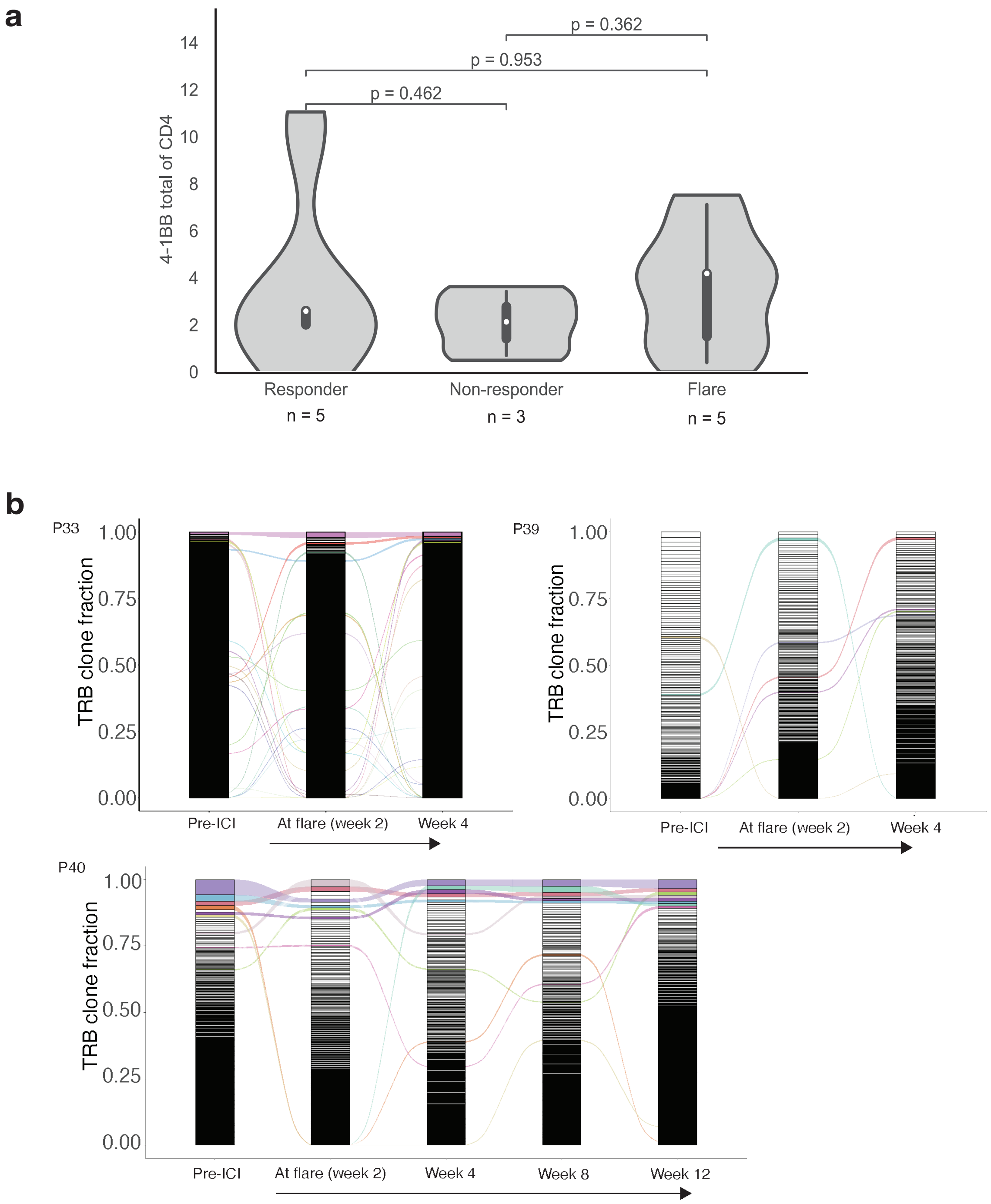

Supplementary Fig.S10. Immune responses in peripheral blood at flare. (a) 4-1BB+ CD4+ T-cells in blood from responders without flare, non-responders and flare. For the box-plots, data are represented as median +/- interquartile range. For statistical significance in comparing responders and non-responders, the Wilcoxon-Mann-Whitney test was used. All p values are 2-sided. **(b)** TRB clonotypes were tracked in 3 patients at baseline, time of flare, and following ICI continuation. Each box represents a distinct TRB clonotype. Clonotypes detected multiple time-points are color-coded and tracked in serial samples over time.

Flow Cytometry Gating Strategy

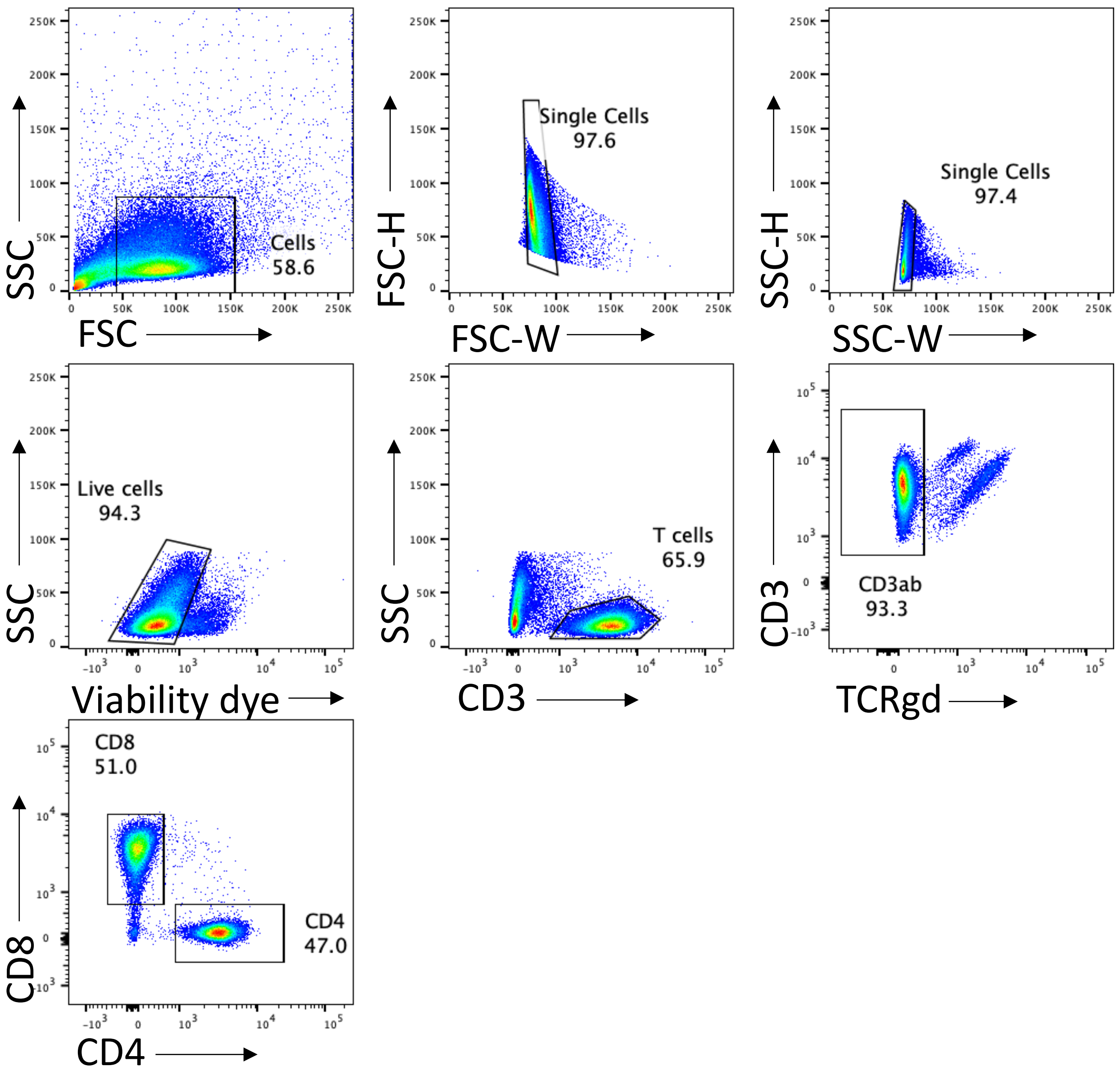

Supplementary Fig. S11. **Gating strategy for flow cytometry utilized in the index study.**

Table S1. Summary of clinical features, outcomes and biomarkers (n=45 tumors in 38 patients)

Patient id	Study ID	Age at biopsy (years)	Sex	Germline	Gene	Tumour Type	Previous treatment	Age at ICI (years)	Disseminated disease	ICI agent	Best radiological response by central review	Progression	Treatment in addition to PD1 ICI	Final Tumor Response	Patient status	Tumor RRD status	Total neoantigens	Total nonsynonymous variants	Total synonymous variants	Total Mutations (SNVs and Indels)	TMB/Mb	Total SNVs	SNVs/Mb	Total Indels	Indels/Mb	Total MS-indels
P01	ICI.42	15	F	CMMRD	PMS2	Colorectal Adenocarcinoma	Surgery + Adjuvant treatment	15.9	No	Nivolumab	Stable disease	No	No	Responder	Alive	MMRD+PPD	24349	8293	3568	17606	352.12	16530	330.6	1076	21.52	832
	ICI.41					Glioblastoma	Surgery + Adjuvant treatment		No	Nivolumab	Complete response	No	No	Responder		MMRD+PPD	22510	7386	3409	16242	324.84	15767	315.34	475	9.5	612
P02	ICI.04	3	M	CMMRD	PMS2	Glioblastoma	Surgery + Adjuvant treatment	4.5	No	Nivolumab	Partial response	Yes	Ipilimumab	Flare and response	Alive	MMRD+PPD	35396	10327	4211	21488	429.76	20881	417.62	607	12.14	342
P03	ICI.11	12.6	F	CMMRD	MSH6	Colorectal Adenocarcinoma	Surgery + Adjuvant treatment	13	Yes	Pembrolizumab	Complete response	No	No	Responder	Alive	MMRD+PPD	28468	16417	6214	34459	689.18	33537	670.74	922	18.44	335
P04	ICI.24	24	M	Lynch (digenic)	PMS2, MSH6	Colorectal Adenocarcinoma	Surgery + Adjuvant treatment	24.2	Yes	Pembrolizumab	Partial response	No	No	Responder	Alive	MMRD	3866	673	0	2820	56.4	1608	32.16	1212	24.24	393
P05	ICI.02	16.7	M	CMMRD	MSH6	Colorectal Adenocarcinoma	Surgery only	17	No	Pembrolizumab	No measurable disease	No	No	Responder	Alive	Not available	Not available	Not available	Not available	Not available	Not available	Not available	Not available	Not available	Not available	Not available
	ICI.01					Anaplastic Astrocytoma	Surgery + Adjuvant treatment		No	Pembrolizumab	No central review	Yes	Ipilimumab	Non-responder		MMRD	3668	786	0	1725	34.5	1673	33.46	52	1.04	1984
P06	ICI.39	16.8	F	Lynch	MLH1	Colorectal Adenocarcinoma	Surgery only	16.8	No	Pembrolizumab	Partial response	No	No	Responder	Alive	MMRD	1172	207	85	497	9.94	409	8.18	88	1.76	123
P07	ICI.22	8.3	M	CMMRD	PMS2	Glioblastoma	Surgery + Adjuvant treatment	9.2	No	Nivolumab	Stable disease	No	No	Responder	Alive	MMRD+PPD	20330	6301	2391	17228	344.56	13769	275.38	3459	69.18	1429
P13	ICI.29	16	M	PPD	POLE	Colorectal Adenocarcinoma	Surgery + Adjuvant treatment	17.5	Yes	Nivolumab	Complete response	No	No	Responder	Alive	MMRD+PPD	21196	9309	2816	18679	373.58	18572	371.44	107	2.14	174
P14	ICI.17	16	M	CMMRD	PMS2	Glioblastoma	Surgery + Adjuvant treatment	16.5	Yes	Nivolumab	Complete response	Yes	Ipilimumab + Re-irradiation + Targeted agent	Responder	Dead	MMRD+PPD	14544	4213	1442	10209	204.18	9229	184.58	980	19.6	332
P15	ICI.37	5	F	CMMRD	PMS2	Glioblastoma	Surgery + Adjuvant treatment	5.5	No	Nivolumab	Partial response	No	No	Responder	Alive	MMRD+PPD	22483	7414	2876	14799	295.98	14351	287.02	448	8.96	668
P16	ICI.08	13.8	M	CMMRD	PMS2	Glioblastoma	Surgery + Adjuvant treatment	14.5	No	Pembrolizumab	Progressive disease	Yes	Re-irradiation	Non-responder	Dead	MMRD+PPD	31690	10331	4134	20793	415.86	20198	403.96	595	11.9	227
	ICI.07					Colorectal Adenocarcinoma	Surgery only		Yes	Pembrolizumab	Partial response	No	No	Flare and response		MMRD+PPD	17070	321	150	6934	138.68	3310	66.2	3624	72.48	1370
P17	ICI.33	30.9	M	PPD	POLE	Glioblastoma	Surgery + Adjuvant treatment	31.2	No	Pembrolizumab	Partial response	No	No	Responder	Alive	MMRD+PPD	29841	11502	4309	23138	462.76	22927	458.54	211	4.22	201
P18	ICI.40	14	M	Lynch	MSH2	Glioblastoma	Surgery + Adjuvant treatment	14.7	No	Nivolumab	Stable disease	No	No	Responder	Alive	Not available	Not available	Not available	Not available	Not available	Not available	Not available	Not available	Not available	Not available	Not available
P19	ICI.43	9	F	CMMRD	MSH6	Glioblastoma	Surgery + Adjuvant treatment	9.2	No	Nivolumab	Stable disease	Yes	No	Responder	Alive	MMRD+PPD	61414	22941	8230	45910	918.2	45596	911.92	314	6.28	480
P20	ICI.19	10.6	M	CMMRD	PMS2	Glioblastoma	Surgery + Adjuvant treatment	11.3	No	Nivolumab	Progressive disease	Yes	Ipilimumab + Re-irradiation + Targeted agent	Non-responder	Dead	MMRD+PPD	16810	9683	3569	19558	391.16	18555	371.1	1003	20.06	75
P21	ICI.46	9.3	M	CMMRD	PMS2	Glioblastoma	Surgery + Adjuvant treatment	9.5	No	Nivolumab	Stable disease	No	No	Flare and response	Alive	MMRD+PPD	12123	4969	1969	10296	205.92	10001	200.02	295	5.9	158
P22	ICI.32	27.6	F	CMMRD	PMS2	Glioblastoma	Surgery + Adjuvant treatment	27.8	Yes	Nivolumab	No central review	Yes	No	Non-responder	Dead	Not available	Not available	Not available	Not available	Not available	Not available	Not available	Not available	Not available	Not available	Not available
P23	ICI.05	6.5	F	CMMRD	PMS2	Glioblastoma	Surgery + Adjuvant treatment	7.1	No	Nivolumab	Partial response	Yes	Ipilimumab	Flare and response	Dead	MMRD+PPD	39642	11522	4187	24186	483.72	23222	464.44	964	19.28	959
P24	ICI.45	7.9	M	CMMRD	PMS2	Glioblastoma	Surgery + Adjuvant treatment	8.2	No	Nivolumab	Complete response	No	No	Responder	Alive	MMRD+PPD	13950	11542	4669	23234	464.68	22590	451.8	644	12.88	199
P25	ICI.44	9.3	M	CMMRD	PMS2	Glioblastoma	Surgery + Adjuvant treatment	9.5	No	Nivolumab	Complete response	No	No	Responder	Alive	Not available	Not available	Not available	Not available	Not available	Not available	Not available	Not available	Not available	Not available	Not available
P26	ICI.23	11.6	F	Lynch	MSH2	Glioblastoma	Surgery + Adjuvant treatment	12.1	No	Nivolumab	Progressive disease	Yes	No	Non-responder	Dead	MMRD	1555	449	191	1284	25.68	871	17.42	413	8.26	195
P27	ICI.35	11	F	CMMRD	MSH6	T Cell Acute Lymphoblastic Lymphoma	Chemotherapy	11.3	Not applicable	Nivolumab	Not applicable	Yes	No	Non-responder	Dead	MMRD	810	289	140	647	12.94	646	12.92	1	0.02	43
	ICI.34					Glioblastoma	Surgery + Adjuvant treatment		No	Nivolumab	Progressive disease	Yes	Ipilimumab	Flare and stopped		MMRD	12254	4904	2820	11771	235.42	11692	233.84	79	1.58	96
P28	ICI.26	10.8	M	CMMRD	MSH2	Colorectal Adenocarcinoma	Surgery only	11.3	No	Nivolumab	No measurable disease	No	No	Responder	Dead	MMRD	2036	409	159	1025	20.5	791	15.82	234	4.68	194
	ICI.25					Glioblastoma	Surgery + Adjuvant treatment		No	Nivolumab	Progressive disease	Yes	No	Flare and stopped		MMRD	1008	257	109	628	12.56	501	10.02	127	2.54	130
P29	ICI.38	16.9	M	Lynch	MLH1	Colorectal Adenocarcinoma	Surgery + Adjuvant treatment	17.2	Yes	Pembrolizumab	Progressive disease	Yes	No	Flare and stopped	Dead	MMRD	5766	608	0	2795	55.9	1305	26.1	1490	29.8	545
P30	ICI.27	12.9	M	CMMRD	PMS2	Acute Myeloid Leukemia	Chemotherapy	13.2	Not applicable	Nivolumab	Not applicable	Yes	No	Non-responder	Dead	MMRD	1404	197	78	523	10.46	411	8.22	112	2.24	45
P31	ICI.09	9.1	F	CMMRD	PMS2	Glioblastoma	Surgery + Adjuvant treatment	9.7	No	Nivolumab	Progressive disease	Yes	No	Flare and stopped	Dead	MMRD+PPD	34116	10624	4020	21090	421.8	20460	409.2	630	12.6	516
P32	ICI.18	11	M	Lynch	PMS2	Ileocecal Signet Cell Carcinoma	Surgery + Adjuvant treatment	12.3	Yes	Nivolumab	Progressive disease	Yes	No	Flare and stopped	Dead	MMRD	6520	664	0	2737	54.74	1531	30.62	1206	24.12	458
P33	ICI.12	2.8	F	CMMRD	MSH2	Glioblastoma	Surgery + Adjuvant treatment	3.1	No	Nivolumab	Progressive disease	Yes	No	Flare and stopped	Dead	MMRD+PPD	19538	8223	3058	17020	340.4	16376	327.52	644	12.88	829
P34	ICI.06	13.2	F	CMMRD	PMS2	CNS Embryonal tumor	Surgery + Adjuvant treatment	14.1	No	Nivolumab	Progressive disease	Yes	No	Non-responder	Dead	MMRD+PPD	9859	2661	1085	5538	110.76	5254	105.08	284	5.68	1226
P35	ICI.14	12.5	F	CMMRD	MSH6	Anaplastic Astrocytoma	Surgery + Adjuvant treatment	13.5	No	Nivolumab	No central review	Yes	No	Non-responder	Dead	MMRD	1499	434	173	858	17.16	787	15.74	71	1.42	96
	ICI.13					Glioblastoma	Surgery + Adjuvant treatment		No	Nivolumab	No central review	No	No	Responder		MMRD+PPD	26175	9797	3956	19902	398.04	19637	392.74	265	5.3	176
P36	ICI.21	13.6	M	CMMRD	MSH6	Anaplastic Astrocytoma	Surgery + Adjuvant treatment	14.4	No	Nivolumab	Progressive disease	Yes	No	Flare and stopped	Dead	MMRD	1406	351	157	744	14.88	669	13.38	75	1.5	101
P37	ICI.10	27.4	F	CMMRD	MSH6	Glioblastoma	Surgery + Adjuvant treatment	28.1	No	Nivolumab	Progressive disease	No	No	Flare and stopped	Dead	MMRD+PPD	54341	18481	7046	37467	749.34	37210	744.2	257	5.14	993
P38	ICI.20	7	M	Lynch	MSH2	Anaplastic Astrocytoma	Surgery + Adjuvant treatment	8.5	No	Nivolumab	Progressive disease	Yes	No	Non-responder	Dead	MMRD	1476	230	127	627	12.54	501	10.02	126	2.52	122
