# Supplementary material for: Genomic predictors of response to PD-1 inhibition in children with germline DNA replication repair deficiency
Source: Nat Med. 2022 Jan 6;28(1):125–35. doi: 10.1038/s41591-021-01581-6 (PMC8799468; doi:10.1038/s41591-021-01581-6)
Supplement: Supplementary file 2 — Reporting Summary [file 41591_2021_1581_MOESM2_ESM.pdf]

nature portfolio

Corresponding author(s): Uri Tabori

Last updated by author(s): Oct 10, 2021

Reporting Summary

Nature Portfolio wishes to improve the reproducibility of the work that we publish. This form provides structure for consistency and transparency in reporting. For further information on Nature Portfolio policies, see our Editorial Policies and the Editorial Policy Checklist.

Statistics

For all statistical analyses, confirm that the following items are present in the figure legend, table legend, main text, or Methods section.

n/a	Confirmed	
☐	☒	The exact sample size (*n*) for each experimental group/condition, given as a discrete number and unit of measurement
☐	☒	A statement on whether measurements were taken from distinct samples or whether the same sample was measured repeatedly
☐	☒	The statistical test(s) used AND whether they are one- or two-sided<br>*Only common tests should be described solely by name; describe more complex techniques in the Methods section.*
☐	☒	A description of all covariates tested
☒	☐	A description of any assumptions or corrections, such as tests of normality and adjustment for multiple comparisons
☐	☒	A full description of the statistical parameters including central tendency (e.g. means) or other basic estimates (e.g. regression coefficient) AND variation (e.g. standard deviation) or associated estimates of uncertainty (e.g. confidence intervals)
☐	☒	For null hypothesis testing, the test statistic (e.g. F, t, r) with confidence intervals, effect sizes, degrees of freedom and P value noted<br>*Give P values as exact values whenever suitable.*
☒	☐	For Bayesian analysis, information on the choice of priors and Markov chain Monte Carlo settings
☒	☐	For hierarchical and complex designs, identification of the appropriate level for tests and full reporting of outcomes
☒	☐	Estimates of effect sizes (e.g. Cohen's *d*, Pearson's *r*), indicating how they were calculated

Our web collection on statistics for biologists contains articles on many of the points above.

Software and code

Policy information about availability of computer code

Data collection	Microsoft Excel 2017 was used for data collection for this study. The IRRDC database uses Microsoft Access 2013 for its registry data collection.
Data analysis	SPSS v.20, R v.3.5 and Python v.2.7 were used for data analysis. Flow cytometry data analysis was performed using FlowJo software 10.8.0. Editing of output figures was done using Adobe Illustrator v.23.0.1. All codes are previously published, publicly available, and are cited exhaustively in 'Methods.'

For manuscripts utilizing custom algorithms or software that are central to the research but not yet described in published literature, software must be made available to editors and reviewers. We strongly encourage code deposition in a community repository (e.g. GitHub). See the Nature Portfolio guidelines for submitting code & software for further information.

Data

Policy information about availability of data

All manuscripts must include a data availability statement. This statement should provide the following information, where applicable:

- Accession codes, unique identifiers, or web links for publicly available datasets
- A description of any restrictions on data availability
- For clinical datasets or third party data, please ensure that the statement adheres to our policy

Data Availability: All data relevant to this work are available at the European Genome Phenome Archive (EGA: https://ega-archive.org/studies/EGAS00001005579; Study EGAS00001005579; Dataset EGAD00001008036) and can be accessed through communication with the corresponding author. Clinical data are in Supplemental Table S1. Reference genomes were downloaded from the publicly available resources at https://genome.ucsc.edu.

Field-specific reporting

Please select the one below that is the best fit for your research. If you are not sure, read the appropriate sections before making your selection.

☒ Life sciences ☐ Behavioural & social sciences ☐ Ecological, evolutionary & environmental sciences

For a reference copy of the document with all sections, see nature.com/documents/nr-reporting-summary-flat.pdf

Life sciences study design

All studies must disclose on these points even when the disclosure is negative.

Sample size	All 45 tumors from 38 patients treated using immune checkpoint inhibition by the IRRDC between May 2015 and March 2019 were included. As this was an observational study, no sample size analysis was performed. However, to the best of our knowledge, this is the largest dataset for children and young adults with germline replication repair deficiency treated using anti-PD1 immune checkpoint inhibition therapy.
Data exclusions	None. As mentioned above, all 45 tumors treated during the aforementioned study period by the IRRDC were included.
Replication	This is an observational registry clinical and biomarker study. Only immunohistochemistry was reviewed blindly and independently scored by two teams of pathologists (CH/NA and OK) centrally with good concordance (Supplementary Fig.S7). Replication was not relevant for other aspects of this study.
Randomization	Randomization is not applicable for an observational study.
Blinding	Blinding is not applicable to an observational study

Reporting for specific materials, systems and methods

We require information from authors about some types of materials, experimental systems and methods used in many studies. Here, indicate whether each material, system or method listed is relevant to your study. If you are not sure if a list item applies to your research, read the appropriate section before selecting a response.

Materials & experimental systems

n/a	Involved in the study	
☐	☐	Antibodies
☒	☐	Eukaryotic cell lines
☒	☐	Palaeontology and archaeology
☒	☐	Animals and other organisms
☐	☒	Human research participants
☒	☐	Clinical data
☒	☐	Dual use research of concern

Methods

n/a	Involved in the study	
☒	☐	ChIP-seq
☐	☒	Flow cytometry
☐	☒	MRI-based neuroimaging

Antibodies

Antibodies used	(A) IMMUNOHISTOCHEMISTRY: PD-L1 (clone28-8, Abcam, 1:500, cat no: ab205921), CD68 (Clone:PG-M1, Dako-OMNIS, ready to use, cat no: GA613), CD8 (Clone: c8/144B, Dako-OMINS, ready to use, cat no: GA623), CD3 (polyclonal rabbit, Dako-OMNIS, ready to use, cat no: GA503), and CD4 (Clone:SP35, Sigma-Aldrich, 1:50, cat no: 104R-1)*.<br>(B) FLOW CYTOMETRY: CD3 (BUV395, clone-UCHT1, BD Biosciences, 1:25, cat no 563546, lot no 0174589), CD4 (Alexa Fluor 700, clone-OKT4, Thermo Fisher Scientific, 1:100, cat no 56-0048-82, lot no 1939044), CD4 (BV605, clone-RPA-T4, BD Biosciences, 1:50, cat no 562658, lot no 8351537), CD8a (PerCP, clone-RPA-T8, BioLegend, 1:50, cat no 301030, lot no B226364), CD8a (Alexa Fluor 700, clone-RPA-T8, Thermo Fisher Scientific, 1:100, cat no 56-0088-42, lot no 428470), CD137 (PE, clone-4B4-1, BD Biosciences, 1:100, cat no 561701, lot no 5093502), TIGIT (PE-Cy7, clone-MBSA43, Thermo Fisher Scientific, 1:50, cat no 25-9500-42, lot no 4330435), Ki67 (FITC, clone-20Raj1, Thermo Fisher Scientific, 1:50, cat no 11-5699-42, lot no 1912018)<br>*The IHCs were done in real-time, often for clinical indications, over the time of this study (5 years; 2015-2019) in the CLIA approved lab at the Hospital for Sick Children. The lot numbers varied according to the time an individual case was stained and hence cannot be individually detailed here.
Validation	All antibodies used are commercially available and were validated by the manufacturer with the details available on respective websites, the links for which are provided as follows:<br>(A) IMMUNOHISTOCHEMISTRY<br>PD-L1: https://www.abcam.com/cancer/pd-l1-28-8-rabmab-knockout-validated-antibody<br>CD68: https://www.agilent.com/en/product/immunohistochemistry/antibodies-controls/primary-antibodies/cd68-(dako-omnis)-76223<br>CD8: https://www.agilent.com/en/product/immunohistochemistry/antibodies-controls/primary-antibodies/cd8-(dako-omnis)-76236<br>CD3: https://www.agilent.com/en/product/immunohistochemistry/antibodies-controls/primary-antibodies/cd3-(dako-omnis)-76197

CD4: https://www.sigmaaldrich.com/CA/en/product/sigma/104r1
(B) FLOW CYTOMETRY:
CD3: https://www.bdbiosciences.com/en-ca/products/reagents/flow-cytometry-reagents/research-reagents/single-color-antibodies-ruo/buv395-mouse-anti-human-cd3.563546
CD4: https://www.thermofisher.com/antibody/product/CD4-Antibody-clone-OKT4-OKT-4-Monoclonal/56-0048-82
CD8a: https://www.biolegend.com/en-us/products/percp-anti-human-cd8a-antibody-4221
CD4: https://www.bdbiosciences.com/en-us/products/reagents/flow-cytometry-reagents/research-reagents/single-color-antibodies-ruo/bv605-mouse-anti-human-cd4.562658
CD8a: https://www.bdbiosciences.com/en-ca/products/reagents/flow-cytometry-reagents/research-reagents/single-color-antibodies-ruo/alexa-fluor-700-mouse-anti-human-cd8.561026
CD137: https://www.bdbiosciences.com/en-eu/products/reagents/flow-cytometry-reagents/research-reagents/single-color-antibodies-ruo/pe-mouse-anti-human-cd137.555956
TIGIT: https://www.thermofisher.com/antibody/product/TIGIT-Antibody-clone-MBSA43-Monoclonal/25-9500-42
Ki67: https://www.thermofisher.com/antibody/product/Ki-67-Antibody-clone-20Raj1-Monoclonal/11-5699-82

Human research participants

Policy information about studies involving human research participants

Population characteristics

Thirty-eight patients who developed 45 cancers were treated with PD-1 inhibitors and followed by the IRRDC study group between May 2015 and March 2019. The PD-1 inhibitor used was either nivolumab (n=34, 75%) or pembrolizumab (n=11, 25%). All patients had germline RRD, diagnosed as constitutional MMRD (n=28, 74%), Lynch (n=8, 21%), or PPD (n=2, 5%) syndromes. Median age at treatment was 12.1 years (range: 3.1-28.1) for patients with constitutional MMRD, and 15.7 years (range: 8.5-43.4) for those with Lynch syndrome (p=0.07). Seven cancer types were included and classified into 3 major groups: central nervous system (CNS) tumours (n=31, 69%; disseminated: 2, 6%), non-CNS solid tumours (n=11, 24%; disseminated: 7, 64%), and haematological malignancies (n=3, 7%). The majority (n=43, 93%) of cancers were progressive/ recurrent after failure of first-line therapy. Three patients with gastrointestinal cancers received ICI directly following surgery; two had synchronous CNS tumours, and one who had metastatic disease. Data cutoff for outcomes was October 2019.

Recruitment

Patients were identified through the International Replication Repair Deficiency Consortium (IRRDC), based at SickKids, Toronto. IRRDC has enrolled >200 patients from 45 countries since 2007. Recruitment was based on patient referral to the international consortium by treating physicians and families, and hence referral bias cannot be completely eliminated. However the consortium is internationally recognized as being led by a team of the leading expert groups in the field. Patients with confirmed or suspected replication-repair deficiency were eligible. Germline diagnosis of constitutional MMRD, Lynch or PPD were confirmed by the IRRDC's genetic counsellor (MA), based on the family history, next-generation panel sequencing of germline samples for MMR and POLE/ POLD1 genes (performed locally or centrally at CLIA-approved laboratories), and immunohistochemical (IHC) staining pattern of the tumour and normal tissues (CH).

Ethics oversight

The Hospital for Sick Children (SickKids) Research Ethics Board.

Note that full information on the approval of the study protocol must also be provided in the manuscript.

Flow Cytometry

Plots

Confirm that:

- [x] The axis labels state the marker and fluorochrome used (e.g. CD4-FITC).
- [ ] The axis scales are clearly visible. Include numbers along axes only for bottom left plot of group (a 'group' is an analysis of identical markers).
- [x] All plots are contour plots with outliers or pseudocolor plots.
- [x] A numerical value for number of cells or percentage (with statistics) is provided.

Methodology

Sample preparation

Viable frozen peripheral blood mononuclear cells were incubated with Fc block (BD Biosciences) prior to staining for surface markers (anti-CD3 - clone UCHT1, anti-CD4 – clone RPA-T4, anti-CD8 – clone RPA-T8, anti-4-1BB– clone 4B4-1, anti-TIGIT – clone MBSA43, anti-Ki67 – clone 20Raj1) and viability dye (eBioscience). Cells were fixed and permeabilized for intercellular staining with the Foxp3 transcription factor staining buffer set (BD).

Instrument

BD LSR Fortessa flow cytometer

Software

FlowJo software 10.8.0

Cell population abundance

Cell population abundance is not applicable for our study as we did not perform any cell sorting as part of our study.

Gating strategy	Cells were first gated on SCC and FSC. Then, doublets were excluded by FSC-H/FSC-W and SSC-H/SSC-W. Live cells were gated based on viability dye negative expression. CD4 and CD8 T cells were gated as CD3 positive and TCRgd negative. A representative gating strategy is shown in Supplementary Fig.S11. From these populations, we evaluated the expression of markers mentioned in the text and shown in figures.

☒ Tick this box to confirm that a figure exemplifying the gating strategy is provided in the Supplementary Information.

Magnetic resonance imaging

Experimental design

Design type	Centralized imaging review was performed as detailed in Methods.
Design specifications	NA
Behavioral performance measures	NA

Acquisition

Imaging type(s)	NA
Field strength	NA
Sequence & imaging parameters	NA
Area of acquisition	NA

Diffusion MRI ☐ Used ☒ Not used

Preprocessing

Preprocessing software	NA
Normalization	NA
Normalization template	NA
Noise and artifact removal	NA
Volume censoring	NA

Statistical modeling & inference

Model type and settings	NA
Effect(s) tested	NA

Specify type of analysis: ☐ Whole brain ☐ ROI-based ☐ Both

Statistic type for inference (See Eklund et al. 2016)	NA
Correction	NA

Models & analysis

n/a	Involved in the study
☒	☐ Functional and/or effective connectivity
☒	☐ Graph analysis
☒	☐ Multivariate modeling or predictive analysis
